# Inhibition of cell surface GRP78 on brain tumors reverses drug resistance and stops cancer stem cell expansion

**DOI:** 10.1016/j.jbc.2026.111146

**Published:** 2026-01-12

**Authors:** Elizabeth Stolarik, Jorryn Zelek, Mariah Thigpen, Gaelle Muller-Greven, Rafaela Besse, Shanuja Jakkala, Anita K. Davidson, Candece L. Gladson, Donald J. Davidson

**Affiliations:** 1 Department of Research and Development, Creative BioTherapeutics, Gurnee, Illinois, USA; 2 Department of Cancer Biology, Cleveland Clinic, Cleveland, Ohio, USA

**Keywords:** brain tumor, glioblastoma, tumor microenvironment, chemoresistance, multidrug transporter, GRP78

## Abstract

Even with the best available treatments for patients with brain cancer, the recurrence rates are nearly 100%. A major cause of recurrence is the expansion of brain tumor stem cells. These stem cells are highly resistant to chemotherapy and radiation and have strong immunosuppressive characteristics. Surface-bound glucose-regulated protein 78 (GRP78) is a survival factor that is essential for tumor stem cell expansion. Cell surface GRP78, compared to endoplasmic reticulum–resident GRP78, has been defined as a stress-induced factor that can cause aggressive growth, chemoresistance, radioresistance, and stem cell expansion in brain tumors. Here, we focused on how cell surface GRP78 induces these protumor effects and how these effects can be reversed. Herein, our data show that surface GRP78 binds and stabilizes a novel transmembrane tyrosine kinase called receptor tyrosine kinase–like orphan receptor-1, an oncofetal factor called Cripto, and a checkpoint protein called programed death-ligand 1 in brain tumor cells. To specifically inhibit only cell surface GRP78, we created and tested several GRP78 inhibitors, CBT100, CBT200, and CBT300. We found that these GRP78 inhibitors regressed pediatric and adult brain tumor neurospheres and eliminated receptor tyrosine kinase–like orphan receptor-1, Cripto, and programed death-ligand 1 expression, leading to tumor cell apoptosis. Cell surface GRP78 inhibition with systemic dosing of GRP78 inhibitors, in preclinical orthotopic brain tumor mouse models, demonstrated significant increases in overall survival and durable regressions in 40 to 70% of treated mice compared to control mice.

Brain cancers are the leading cause of disease-related death in children and adults under the age of 40 years (1). Furthermore, high-grade glioma, a very aggressive type of brain cancer, comprises over 80% of malignant brain tumors in adults (1, 2). Even with aggressive treatments that include maximal surgical resection, stereotactic radiosurgery, whole-brain radiation therapy (WBRT), chemotherapy, molecularly targeted therapeutics, and immunotherapies, the recurrence rates for many brain cancers are nearly 100% (3, 4, 5). These recurrent brain cancers are frequently drug-resistant, which leads to a dismal 2-year median survival rate for children and adults. Specifically, childhood brain cancer types, diffuse intrinsic pontine glioma (DIPG), and glioblastoma have a median survival rate of less than 1 year (6, 7). Despite new treatment technologies, new therapeutic targets, and advanced diagnostic methods for brain cancer patients over the past decade, major challenge of drug resistance leading to tumor recurrence persist. Currently, there are no available medications that can reverse drug resistance and eliminate brain cancer recurrence.

The inevitable relapse of brain cancer results from the escape and expansion of drug and immune resistant glioma stem cells (GSCs) (7). These GSCs have been shown to have a codependent and synergistic relationship with the brain tumor microenvironment (TME) (8, 9, 10). Recently, it has been shown that the hypoxic, low glucose TME is also important in early and in late stages of glioma tumor development, growth, and drug resistance (10, 11). This crosstalk between metabolism and drug resistance in the brain TME remains largely undefined.

A novel survival factor, glucose-regulated protein 78 (GRP78) has been shown to be highly upregulated under stressed TME conditions (12, 13, 14). In normal cells, GRP78 is an endoplasmic reticulum (ER) resident protein that exists intracellularly to facilitate folding of proteins and targeting misfolded proteins for ER-associated degradation (15, 16, 17). However, in tumor and stromal cells during stress, GRP78 is released from the ER and translocated to the cell surface (16, 18, 19). Thus surface-bound GRP78 has been shown to be highly expressed in breast, lung, brain, prostate, colon, multiple myeloma, renal, and ovarian cancers compared to normal tissues (16, 19). Studies also show that cell surface GRP78 modulates drug resistance in pancreatic (gemcitabine) (20), breast (doxorubicin, tamoxifen) (21), prostate (paclitaxel, bortezomib, rapamycin) (22, 23, 24), lung (cisplatin) (25), hepatocellular (5-FU) (26), multiple myeloma, and brain cancers (27, 28). Increased surface-bound GRP78 expression on brain tumors has been shown to correlate with later stage, resistance to radiation and chemotherapies, increased metastases, and aggressiveness (16, 19, 27).

Previously, we and others have demonstrated that inhibition of surface GRP78 on brain tumor cells by K5 leads to tumor cell apoptosis and inhibition of angiogenesis (12, 13). Perri *et al.* published that when U87 glioblastoma cells were transduced to express this GRP78 inhibitor, K5, and implanted in the forebrain of nude mice; the expressed K5 significantly suppressed glioma growth and promoted long-term survival of mice for 120 days (28). The authors then showed that the K5-expressing tumor cells in immune-competent mice significantly recruited CD3 T cells including, CD4, CD8, and NKT cells. This recruitment led to a >300-day extended survival in mice (29). These studies suggest that inhibition of surface-bound GRP78 can reverse immune suppression and drug resistance. However, none of these studies have demonstrated that a systemically dosed, specific cell surface GRP78 inhibitor directly leads to brain tumor regression. However, the mechanism by which cell surface GRP78 induces resistance and immunosuppression remains unclear. In this study, we showed that elimination of cell surface GRP78 with our novel and specific cell surface GRP78 inhibitor reduced the expression of oncofetal proteins receptor tyrosine kinase–like orphan receptor-1 (ROR1), Cripto, and programed death-ligand 1 (PD-L1), leading to increased chemosensitivity, reduced immune suppression, and elimination of stem cell expansion.

## Results

### Extracellular GRP78 binding increases resistance to doxorubicin of brain tumor cells

**Figure 1:**
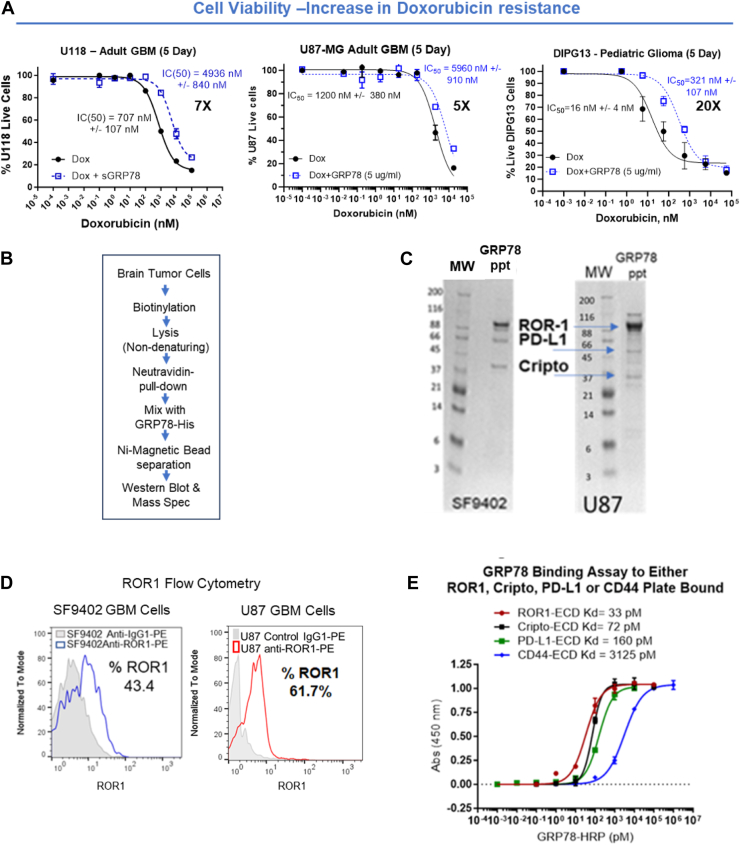
**Extracellular GRP78 induces drug resistance through binding to cell surface ROR1, Cripto-1, and PD-L1.***A,* five-day cellular viability of U118, U87, and DIPG13 glioma cells with and without extracellular GRP78 (5 μg/ml) and under treatment at different concentrations of doxorubicin was measured with a live-cell CCK-8 assay. *B,* pediatric GBM SF9402, adult GBM U87, cell membrane proteins were biotinylated and solubilized using a nondenaturing membrane isolation kit and GRP78-bound proteins were eluted and analyzed by SDS-PAGE and mass spectrometry analysis. *C,* PAGE analysis of cell surface GRP78-binding proteins. Mass spectrometry analysis determined the bands were ROR1, PD-L1, and Cripto. MW. Molecular Weight Standards in kDa, GRP78 ppt = Surface proteins bound to GRP78. *D,* flow cytometry analysis of ROR1 expression on pediatric GBM SF9402 (43.4%) and adult GBM U87 (61.7%) cells. *E,* extracellular domains of ROR1, Cripto-1, PD-L1, and CD-44 were tested for GRP78 binding using a direct ELISA method. Kd = ROR1<Cripto < PD-L1<<CD44. The results in (*A*), (*D*), and (*E*) are the average of ≥3 technical replicates. In XY plots, each point represents the mean value of four replicates ± SD. CCK-8, Cell Counting Kit-8; GBM, glioblastoma multiforme; GRP78, glucose-regulated protein 78; PD-L1, programed death-ligand 1; ROR1, receptor tyrosine kinase–like orphan receptor-1; DIPG, diffuse intrinsic pontine glioma.

To ascertain whether extracellular GRP78 can bind directly to tumor cells to increase chemoresistance and survival, cell viability assays with doxorubicin were performed with and without 5 μg/ml of extracellular GRP78. The 5 μg/ml concentration of GRP78 was derived from publications that show extracellular GRP78 circulates in the blood at 5 to 15 μg/ml, 10 to 15 μg/ml, 1 to 5 μg/ml, and 1 to 2 μg/ml in lung, ovarian, multiple myeloma, and glioblastoma multiforme (GBM) patients, respectively (30, 31, 32, 33). Doxorubicin was chosen for combination therapy since it has recently been mixed with liposomes and shown to cross the blood–brain barrier (34). As such, we added 5 μg/ml of GRP78 to several different types of brain tumor cells for a 5-day viability assay with various concentrations of doxorubicin (Fig. 1*A*). GRP78 is a full-length protein that has been shown to be correctly folded by antibody binding and has active ATPase activity but does not have an ATP or ADP bound in the ATPase domain (determined by the manufacturer). Extracellular GRP78 increased tumor cell resistance to doxorubicin by 5× in U87MG cells, 7× for U118 cells and 20× in DIPG13 cells in a 5-day viability assay (Fig. 1*A*).

### GRP78 binds to surface receptors, ROR1, Cripto, and PD-L1, on brain tumor cells

To identify how extracellular GRP78 binds to cancer cell surfaces and induces chemoresistance, we used C-terminal His-tagged GRP78 in pull-down experiments (Fig. 1*B*). We chose pediatric SF9402 DIPG stem cells and U87 adult GBM cells because they have H3 and IDH WT signatures that have been shown to be more chemotherapy resistant than IDH mutant or H3K27M mutant DIPG/GBM cells (Table 1) (35). From these pull downs, we identified a novel GRP78-binding receptor called ROR1 and two known cell surface GRP78-binding proteins, Cripto and PD-L1 (Fig. 1*C*) (36, 37). To confirm that ROR1 is on the surface of brain tumor cells (SF9402, U87), we used flow cytometry to analyze the surface expression of ROR1. As shown in Figure 1*D*, more than half of the cells from both cell lines expressed ROR1 when 5 μg/ml of extracellular GRP78 was added. As ROR1 has not been previously shown to bind to GRP78, we compared its affinity (Kd) for binding to GRP78 to that of Cripto (36), PD-L1 (37), and CD44 (38). In Figure 1*E* of each domain was compared (binding affinity) to GRP78. The extracellular domains (ECDs) of Cripto, PD-L1, and ROR1 bound very tightly, with affinities in the picomolar range, whereas CD44 bound to GRP78 was approximately 100-fold weaker, with an affinity in the nanomolar range.

### ROR1, PD-L1, and Cripto are highly expressed on patient brain tumors with proximity to cell surface-bound GRP78

**Figure 2:**
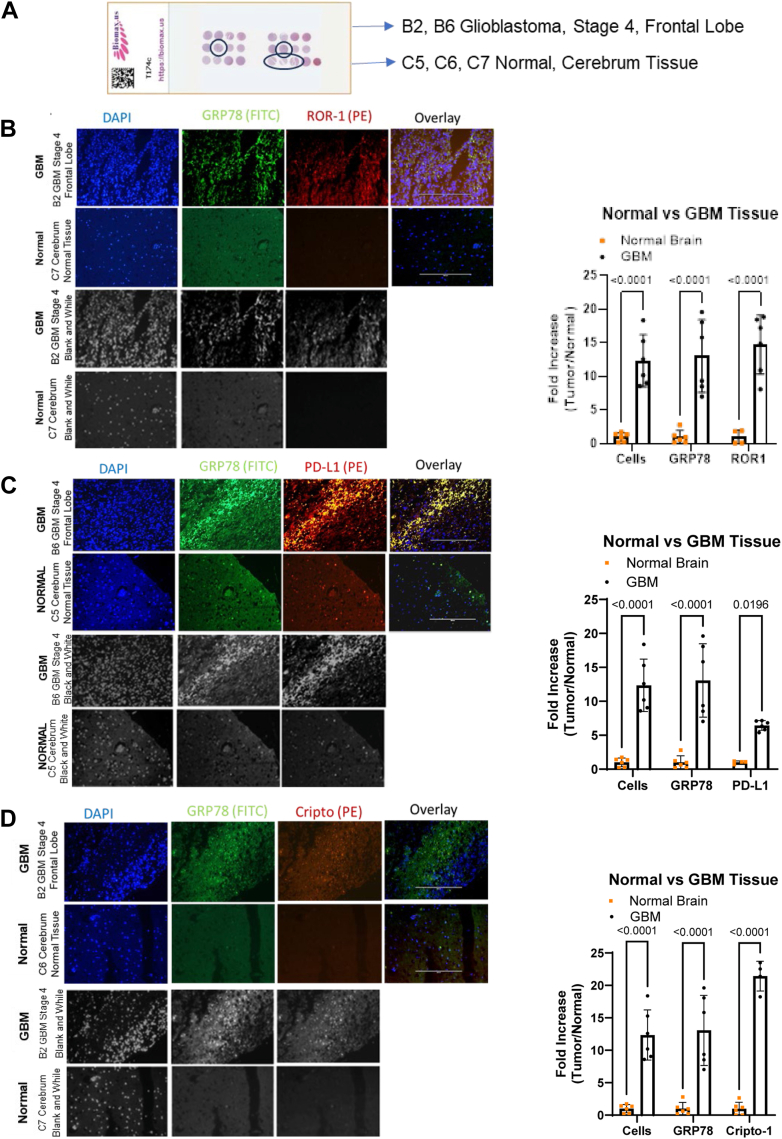
**Cell surface GRP78 colocalizes with ROR1, Cripto, and PD-L1 on late-stage patient GBM tissues but not on normal brain cerebrum tissues.***A,* three brain tissue microarrays with patient core tissue samples of GBM and normal tissues (#T174T from Biomax.us.) were stained for GBM markers. *B,* one microarray was stained with 4′,6-diamidino-2-phenylindole (DAPI) (*blue*-DNA), and antibodies to cell surface GRP78 (*green*-FITC), ROR1 (*red*-PE). The last column is an overlay of all three stains to show colocalization. Cores B2 (stage 4 GBM) and C7 (normal cerebrum) are pictured. *C,* a second microarray was stained with DAPI (*blue*-DNA), and antibodies to cell surface GRP78 (*green*-FITC), PD-L1 (*red*-PE). The last column is an overlay of all three stains to show colocalization. Cores B6 (stage 4 GBM) and C5 (normal cerebrum) are pictured. *D,* a third microarray was stained with DAPI (*blue*-DNA), and antibodies to cell surface GRP78 (*green*-FITC), PD-L1 (*red*-PE). The last column is an overlay of all three stains to show colocalization. Cores B6 (stage 4 GBM) and C5 (normal cerebrum) are pictured. Scale bars represent 400 μm (10×). All images are from the same magnification of (10×) 400 μm. GBM, glioblastoma multiforme; GRP78, glucose-regulated protein 78; PD-L1, programed death-ligand 1; ROR1, receptor tyrosine kinase–like orphan receptor-1.

To compare patient brain tumor tissues with normal brain tissue expression (Fig. 2*A*) of ROR1, PD-L1, Cripto, and cell surface GRP78, microarray slides were stained with 4′,6-diamidino-2-phenylindole (DAPI) (blue DNA), anti-GRP78-FITC (green), and **a)** anti-ROR1-phycoerythrin (PE) (red) (Fig. 2*B*), **b)** anti-PD-L1-PE (red) (Fig. 2*C*), or **c)** anti-Cripto-PE (red) (Fig. 2*D*). From these immunofluorescence staining analyses, we can now show that ROR1, PD-L1, and Cripto colocalize near cell surface GRP78, as indicated by the yellow staining color in the overlay slides, validating our results from our GRP78 pull-down experiments and suggesting the binding of these proteins with cell surface GRP78 in patient brain tumor tissues. We also showed that GRP78, ROR1, PD-L1, and Cripto were more highly expressed in patient brain tumor tissues than in normal brain tissues (Fig. 2, *A*–*D*).

### ROR1 binds to GRP78 through its kringle domain

**Figure 3:**
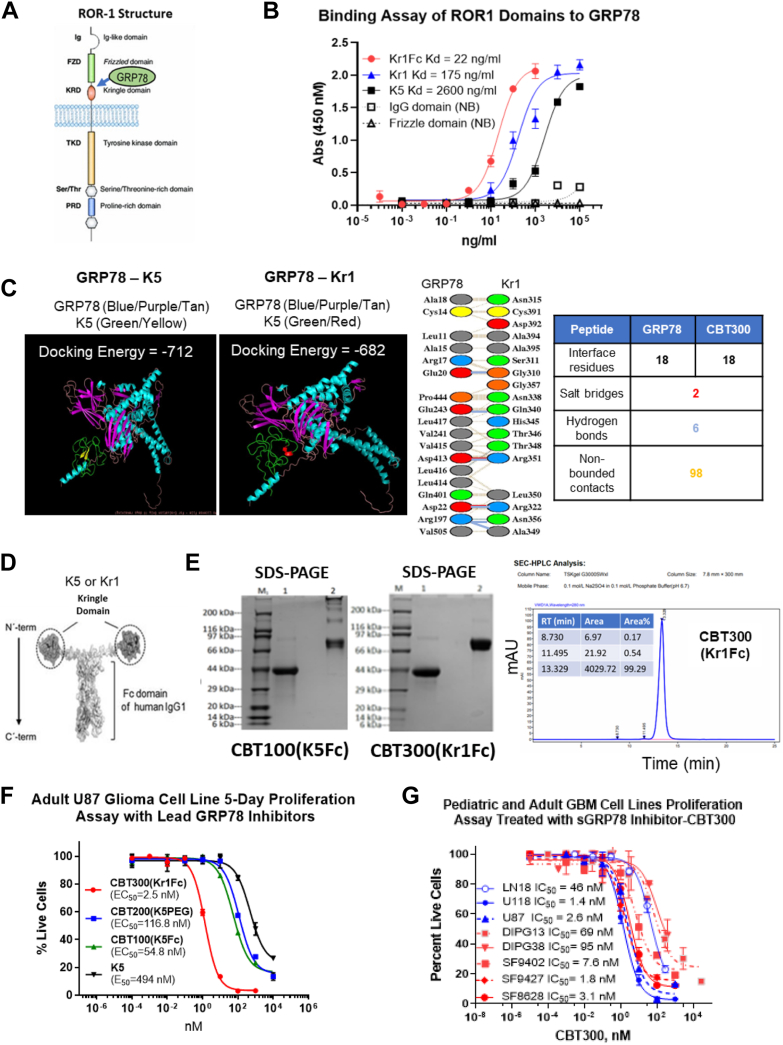
**CBT300’s ROR1 kringle domain (Kr1) domain binds to GRP78 and inhibits tumor cell proliferation.***A,* secondary structure of ROR1 showing where GRP78 is estimated to bind to ROR1 kringle domain. *B,* binding of individual ROR1 Extra Cellular Domains (Ig, FZD, KRD) and other kringle GRP78 binding domains (Kr1, Kr1Fc, K5) to GRP78. *C, in silico* docking of K5 kringle domain or the ROR1 kringle(Kr1) domain with GRP78. The lowest free energy of binding is shown for each of the kringle domains to GRP78. ROR1 kringle (Kr1) shows strong binding with two salt bridges, six hydrogen bonds and 98 nonbonded contacts to GRP78. *D,* graphic depiction of CBT100 (K5Fc) or CBT300 (Kr1Fc). *E,* SDS-PAGE gels of CBT100 and CBT300 showing reduced (#1) and nonreduced (#2) lanes. For both SDS-PAGE gels, M = molecular weight standards, #1 = reduced CBT100 (K5Fc) or CBT300 (Kr1Fc) and #2 = nonreduced CBT100 (K5Fc) or CBT300 (Kr1Fc). Size-exclusion analysis of CBT300 showing > 99% purity after protein A purification. *F,* five-day cell viability assay of U87 GBM cells treated with various concentrations of K5, CDT300 (Kr1Fc) CBT200 (K5PEG), and CBT100 (K5Fc) in triplicate wells. *G,* cell viability in a 5-day assay of four pediatric DIPG stem cell lines and two pediatric GBM stem cell lines under treatment with various concentrations of CBT300. The number of live cells was measured with a CCK-8 reagent. All dose response curves were fitted with a 3-parameter curve fits calculating the IC50 value. CCK-8, Cell Counting Kit-8; GBM, glioblastoma multiforme; GRP78, glucose-regulated protein 78; PD-L1, programed death-ligand 1; ROR1, receptor tyrosine kinase–like orphan receptor-1; DIPG, diffuse intrinsic pontine glioma; CBT, Creative BioTherapeutics.

In analyzing ROR1’s ECD structure (Fig. 3*A*) revealed that ROR1 has a kringle domain. The kringle domain is composed of approximately 80 amino acids with three disulfide bonds. We previously reported that the fifth kringle domain (K5) from human plasminogen binds to cell surface GRP78 (12, 13), and since the ROR1 kringle domain is more than 50% homologous to the K5 kringle domain, we predicted that the ROR1 kringle domain would also bind to GPR78. To determine whether this prediction was true, we tested the ROR1 Ig (Glu39–Gly151), Frizzled (Glu165-Asp305), and Kringle (Asn308–Asp395) domains’ (ECDs) bind GRP78. We found that the only ECD from ROR1 that binds to GRP78 was the kringle domain, Kr1 (Kd = 17.5 nM), which binds 15-fold more tightly than K5 (Kd = 260 nM) (Fig. 3*B*). To further validate this finding, we used the *in silico* docking program ClusPro 2.0 to predict if GRP78 and the ROR1 kringle domain bind (39). In Figure 3*C*, we compared the docked binding energy of GRP78-K5 with GRP78-Kr1. The GRP78-K5 lowest binding energy was −717 kcal/mol, which is comparable to the GRP78-Kr1 binding energy of −682 kcal/mol. The amino acid interactions between GRP78 and the ROR1 kringle domain included 106 interactions with two salt bridges, six hydrogen bonds, and 98 nonbonded contacts. These docking data and the direct binding ELISA data demonstrated that the ROR1 kringle and GRP78 bind.

### Development of a novel extracellular and surface GRP78 inhibitor, Kr1Fc (CBT300)

We previously determined that K5 was a poor drug candidate due to its short half-life in mice and dogs (<2 h, subcutaneous (sc)) (40). Since Kr1 binds to GRP78 15-fold tighter than K5, we created a novel kringle fusion protein with a human IgG1 Fc domain (Fig. 3*D*). The addition of an IgG Fc domain to peptides has been shown to increase plasma half-life significantly (41). Our Kr1Fc fusion protein (CBT300) and K5Fc fusion protein (CBT100) were expressed in CHO cells at our lab and at GenScript. CBT300 was also expressed using Thermo Fisher Scientific and Profacgen. The CBT100 and CBT300 were then purified over a protein A column using methods previously described (42). The binding of CBT100 and CBT300 to benzamidine agarose as a second purification step ensured that the kringle domain was properly folded (42). The purity of CBT100 and CBT300 by SDS-PAGE gel analysis showed that both fusion proteins were at least 95% pure (Fig. 3*E*). However, CBT100 (K5Fc) showed higher molecular weight aggregates that were not observed in CBT300 SDS-PAGE analysis. Size-exclusion HPLC analysis showed that CBT300 (Kr1Fc) purified on a protein A column was over 99% pure. Kr1Fc (CBT300) was added to a 96-well plate and various concentrations of GRP78-horseradish peroxidase (HRP) were added for binding affinity analysis. As shown in Figure 3*B*, GRP78 bound to Kr1Fc (CBT300) at a concentration of 22 ng/ml.

### CBT300 is more potent than K5, CBT100 (K5Fc), and CBT200 (K5PEG) at reducing viability of an adult brain tumor cell line

To evaluate the anticancer effects of CBT300 (Kr1Fc) compared to other GRP78 inhibitors, an adult GBM cell line, (U87) was selected because of its inherent resistance to chemotherapy (43, 44) and was used in a 5-day viability assay. The IC50 values were determined for each inhibitor. CBT300 was shown to be a lead inhibitor of adult GBM tumor cell viability, with an IC50 value of 2.5 nM (Fig. 3*F*). CBT300 (Kr1Fc) was 195-fold more potent than K5, 45-fold more potent than CBT200 (K5PEG), and 20-fold more potent than CBT100 (K5Fc).

### CBT300 decreases cell viability in pediatric and adult patient-derived brain tumor cells

**Figure 4:**
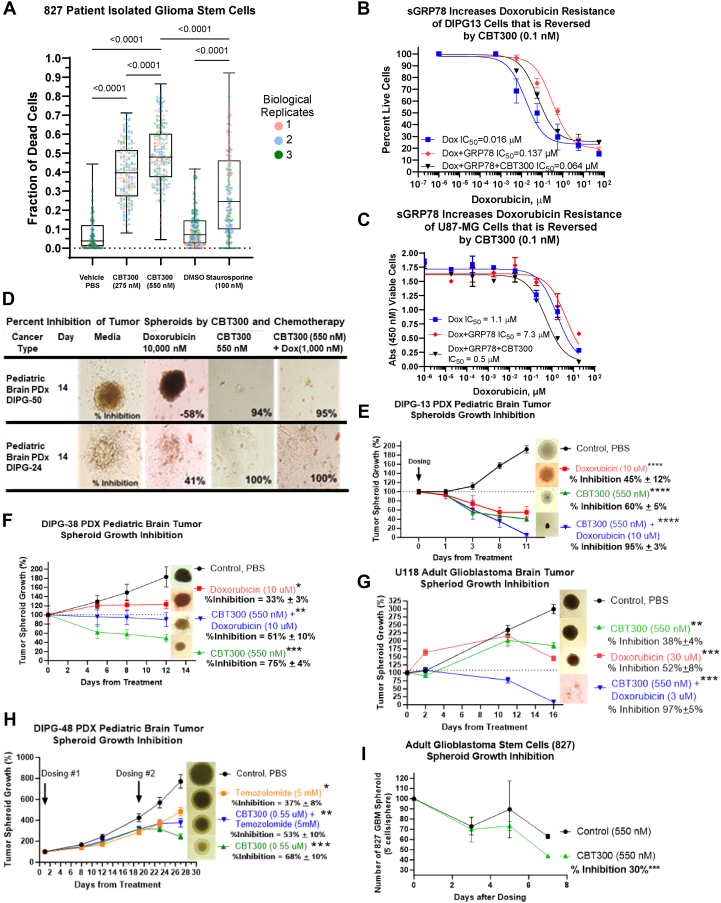
**CBT300’s anticancer efficacy increases cell death in 2D assays and regresses tumor brain tumor spheroids in 3D assays with patient derived adult and pediatric glioma stem cells.***A,* CBT300 significantly increased cell death of patient derived glioma stem cells (827) grown on laminin. Three replicates of 60 cell count each were used with trypan blue reagent to determine dead cells. *B* and *C,* cell viability assays with pediatric and adult glioma cells. Cells were tested in triplicate repeats with doxorubicin with or without GRP78 (5 μg/ml) and with or without CBT300 (0.1 nM). Extracellular GRP78 increases resistance to doxorubicin and CBT300 reverses this resistance in cell viability assays. *D,* Pediatric Diffuse Intrinsic Ponte Glioma stem cells were seeded at 10,000 cells per well in 96 U-bottom well ultra low attachment (ULA) plates and cultured as a spheroid for 14 days with either medium, doxorubicin (10 μM), CBT300 (550 nM), or CBT300 (550 nM) + doxorubicin (1 μM). Representative images of DIPG-50 and DIPG-24 spheroids are shown. *E–H,* pediatric and adult glioma cells were seeded in ULA plates in triplicate wells with the treatments listed. Pictures of spheroids were taken at various times, and the size of the spheroids was measured by Image J (https://imagej.net). Spheroid regression/growth curves points are a mean of four replicates with standard deviation shown. The final point of measurements with a representative spheroid picture was shown with treatment regime and percent inhibition compared to the Control. Standard *p* values (∗*p* < 0.5, ∗∗*p* < 0.01, ∗∗∗*p* < 0.001, and ∗∗∗∗*p* < 0.0001) with respect to control were calculated by two-tailed unpaired *t* test for final measurements. *I*, patient derived adult 827 glioma stem cells were plated at 30,000 cells per well in 96-flat well plates coated with laminin. Cells were treated with either PBS control or CBT300 (550 nM) for 7 days. Spheroids containing more than or equal to five cells were counted on the days shown. Percent inhibition of spheroid numbers was calculated at the final time point. Standard *p* value (∗∗∗*p* < 0.001) with respect to control was calculated by two-tailed unpaired *t* test for final measurements. The data points are an average of 3–4 replicates. CCK-8, Cell Counting Kit-8; GBM, glioblastoma multiforme; GRP78, glucose-regulated protein 78; PD-L1, programed death-ligand 1; ROR1, receptor tyrosine kinase–like orphan receptor-1; DIPG, diffuse intrinsic pontine glioma; CBT, Creative BioTherapeutics.

To determine whether our lead compound, CBT300, could induce apoptosis in pediatric patient-derived brain tumor cells, we tested CBT300 in the pediatric GBM cell lines SF9402 and SF9427, and the pediatric DIPG cell lines SF8628, DIPG13, DIPG38, and DIPG50. These cell lines were chosen because they were resistant to chemotherapy and attached to tissue-coated plates for 2D viability assays (Table I). Cell viability was assessed using a 5-day Cell Counting Kit-8 (CCK-8) live-cell assay with a dose-response curve of CBT300 (Fig. 3*G*). As shown, CBT300 significantly decreased all tested pediatric DIPG cells and GBM cell viability at IC(50) at low nanomolar concentrations. Because brain tumor cells attached to tissue-coated plastic in a 2D method are not considered stem-like, we wanted to determine whether CBT300 induces apoptosis in patient-derived GBM stem cells (827) grown on laminin. As shown in Figure 4*A*, CBT300 significantly increased cell death of patient-derived GSCs. Three replicates of 60 cell counts were used with the trypan blue reagent to determine dead cells. A significant 50% increase in cell death for GSCs were observed in the CBT300-treated fraction compared to the controls.

**Table 1:** Adult and pediatric glioma characteristics

Patient-derived glioma line				Mutation status						Treatments
Cell line	Type	Age	Sex	Histone	ACVR1	TP53	IDH	NF1	PDGF-RA	(XRT = radiation)
U87	Glioblastoma	Adult	Male	WT		WT	WT			
LN18	Glioblastoma	Adult	Male	WT		MUT	WT			
U118	Glioblastoma	Adult	Male	WT			WT			
827	Glioblastoma	Adult	Male	WT			WT			
SF9402	Glioblastoma	Pediatric^c^	Female	WT			WT			
SF9427	Diffuse HGG^a^	Pediatric^c^	Female	WT			WT			
SF8628	DMG^b^	Pediatric^c^	Female	H3.3K27M	WT	WT	WT	WT	WT	
DIPG13	DMG^b^	Pediatric^c^	Female	H3.3K27M		Mut	WT			XRT
DIPG21	DMG^b^	Pediatric^c^	Male	H3.1K27M	Mut		WT	Mut	Mut	XRT+MK1775
DIPG24	DMG^b^	Pediatric^c^	Female	H3.3K27M						XRT
DIPG38	DMG^b^	Pediatric^c^	Female	H3.3K27M	WT					XRT, Avastin, U.Pitt Vaccine, vorinostat, trametinib, palbociclib, everolimus
DIPG48	Diffuse DMG^b^	Pediatric^c^	Male	WT						XRT, monterrey interatrial therapy
DIPG50	DMG^b^	Pediatric^c^	Female	H3.3K27M	WT	WT	WT	WT	WT	None

PDGF, platelet-derived growth factor; DIPG, diffuse intrinsic pontine glioma; WT = wild type; Mut = mutant.

a High Grade Glioma H3-WT, IDH-WT.

b Diffuse Midline Glioma, H3 K27-altered.

c Pediatric = ages 3 to 14.

Since it has been shown that extracellular GRP78 circulates in several types of cancer patients at approximately 4 to 10 μg/ml (30, 31, 32, 33), we wanted to determine whether extracellular GRP78 added to brain tumor cells could directly induce drug resistance, and whether CBT300 could reverse this resistance. In Figure 4, *B* and *C*, doxorubicin alone decreases adult U87-MG and pediatric DIPG13 cell viability with IC(50)s at 1.1 μM and 0.016 μM, respectively. When 5 μg/ml of extracellular GRP78 is added to the cells, the IC(50) for doxorubicin is 4- to 8-fold weaker at 4.7 μM and 0.137 μM, respectively. Finally, when a low dose of CBT300 (0.1 nM) was added along with extracellular GRP78 and doxorubicin to the cells, the IC(50) values for doxorubicin were greater than 10-fold more potent in both cells (Fig. 4, *B* and *C*).

### CBT300 alone and in combination with doxorubicin significantly regresses established patient-derived glioma neurospheres by over 90%

Glioma cells grown in 3D neurospheres or spheroids more closely resemble tumors *in vivo* with zones of hypoxia and poor diffusion (43, 44). Since we have shown that extracellular GRP78 is expressed in the TME and can increase tumor drug resistance, we added extracellular GRP78 (5 μg/ml) and tested CBT300’s inhibition of pediatric and adult glioma cells grown in neurospheres (Fig. 4, *D*–*I*). After the cells were grown for 2 to 3 days, doxorubicin (10 μM or 30 μM), CBT300 (550 nM), or doxorubicin (1 μM or 3 μM) plus CBT300 (550 nM) was added to the spheres. CBT300 alone significantly regressed pediatric DIPG neurospheres between 60% and 100% (Fig. 4, *D*–*F*, *H*). The results for pediatric and adult GSC neurospheres are detailed in Figure 4, *D*–*I*. Doxorubicin alone inhibited U118 spheres by 52% (*p* < 0.0001). However, the combination of CBT300 and low-dose doxorubicin resulted in a significant synergistic spheroid regression of 97% (*p* < 0.0001) (Fig. 4*G*). For patient-derived GSC827 cells, CBT300 at 550 nM showed a significant 30% regression in the number of spheroids in 7 days (Fig. 4*I*).

### CBT300 eliminates GRP78, ROR1, Cripto, and PD-L1 expression on glioma cells

**Figure 5:**
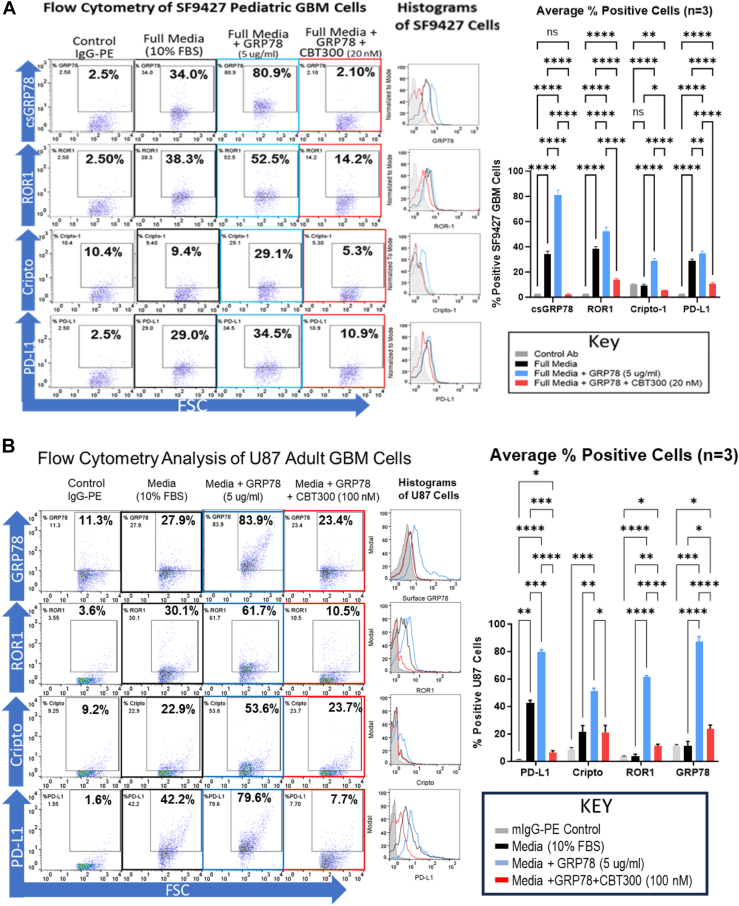
**CBT300 reduces expression of cell surface GRP78, ROR1, Cripto, and PD-L1.***A,* pediatric SF9427 DIPG cells (50,000) were incubated with either media, media+extracellular GRP78 (5 μg/ml), or media+extracellular GRP78 (5 μg/ml)+CBT300 (100 nM) for 72 h. Cells were then stained with mAbs for cell surface GRP78(csGRP78 nonpermeabilized cells), ROR1, Cripto, and PD-L1. Flow cytometry analysis was used on a Guava PCA to determine surface expression of listed cell surface proteins. The average for the percent positive cells is reported as the mean with SD from three replicate assays. *B,* adult GBM cells (50,000) were incubated with either media, media+extracellular GRP78 (5 μg/ml), or media+extracellular GRP78 (5 μg/ml)+CBT300 (100 nM) for 72 h. Cells were then stained with mAbs for cell surface GRP78(csGRP78 nonpermeabilized cells), ROR1, Cripto, and PD-L1. Flow cytometry analysis was used on a Guava PCA to determine surface expression of listed cell surface proteins. The average for the percent positive cells is reported as the mean with SD from three replicate assays. Studies were performed with three independent replicates. The average mean and SD for each set and marker are shown. Significance was determined between groups by two-way ANOVA analysis with post hoc Tukey’s test using GraphPad Prism 10.1.2. ∗*p* < 0.05, ∗∗*p* < 0.01, ∗∗∗*p* < 0.001, and ∗∗∗∗*p* < 0.0001. CCK-8, Cell Counting Kit-8; GBM, glioblastoma multiforme; GRP78, glucose-regulated protein 78; PD-L1, programed death-ligand 1; ROR1, receptor tyrosine kinase–like orphan receptor-1; DIPG, diffuse intrinsic pontine glioma; CBT, Creative BioTherapeutics.

We now know that extracellular GRP78 binds to the kringle domain of ROR1 on the surface of brain tumor cells and speculate that ROR1 is stabilized similarly to how GRP78 stabilizes Cripto and PD-L1 on tumor cell surfaces (36, 37, 38, 39, 40, 41, 42, 43, 44, 45, 46) (www.cellsignal.com). To test this theory, we used flow cytometry analysis of pediatric GBM cells (SF9427), and adult GBM cells (U87) with extracellular GRP78 (5 μg/ml) with/without CBT300 added to the cell lines. In Figure 5, *A* and *B*, the addition of 5 μg/ml extracellular GRP78 for 72 h (Figs. S1 and S2) upregulated the surface expression of GRP78 by 2.4-fold in glioma cells. With the addition of CBT300 for 24 h, GRP78’s surface expression is eliminated by 97% in SF9427 cells and 73% in U87 cells. This significant decrease in surface GRP78 expression resulted in a 73% %decrease in ROR1 expression in SF9427 cells and 83% in U87 cells. The same pattern was observed for the oncofetal protein Cripto and checkpoint protein PD-L1, where their expression was upregulated by extracellular GRP78 addition and downregulated when CBT300 was added to glioma cells.

### CBT300 enhances doxorubicin internalization in pediatric and adult glioma cells

**Figure 6:**
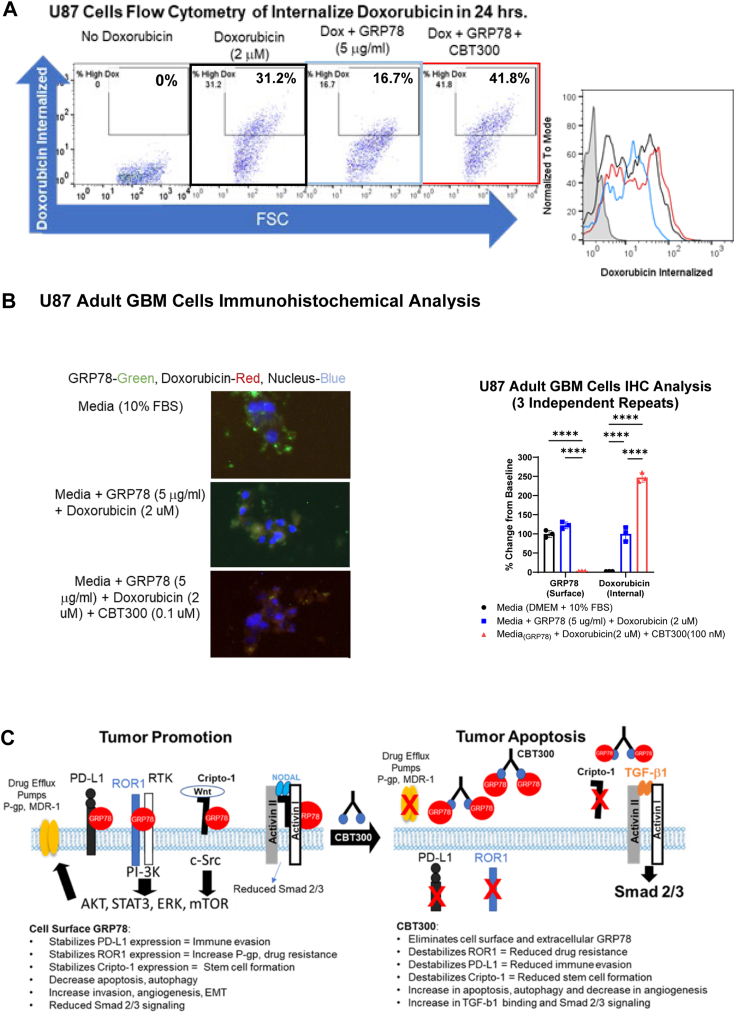
**CBT300 reduces expression of cell surface GRP78 leading to increased doxorubicin internalization in glioma cells.***A,* U87 cells (20,000) were seeded in quadruplicate wells of tissue coated 96-well plates with either media (negative control – no doxorubicin), media (positive control – doxorubicin (2 μm)), media+GRP78 (5 μg/ml), or media+GRP78 (5 μg/ml)+CBT300 (100 nM) for 48 h. Doxorubicin (2 μM) was then added to all the wells except for the negative control wells. 24 h after adding doxorubicin to the cells, cells were washed and flow cytometry analysis for red doxorubicin fluorescence was performed. *B,* U87 cells (3,000) were plated in four well chamber slides and allowed to attach overnight. Each chamber was treated with either media, media+GRP78 (5 μg/ml), or media+GRP78 (5 μg/ml)+CBT300 (100 nM) for 48 h. Doxorubicin (2 μM) was then added to each chamber and incubated for 24 h. Cells/chambers were then washed and stained with a mAb-FITC (*green*) to the C-terminal domain of GRP78, and 4′,6-diamidino-2-phenylindole (*blue*) to the DNA. Doxorubicin, which has a natural red fluorescence, was also measured for each treatment. A chart of three independent repeats was compiled for each marker, cell surface GRP78, and increase in doxorubicin internalized. Studies were performed with three independent replicates. The average mean and standard deviation for each set and marker are shown. Significance was determined between groups by two-way ANOVA analysis with post hoc Tukey’s test using GraphPad Prism 10.1.2. *∗p < 0.05, ∗∗p < 0.01, ∗∗∗p < 0.001,* and *∗∗∗∗p < 0.0001. C,* the proposed mechanism of action of CBT300’s inhibition of surface expressed GRP78 leading to tumor regression and apoptosis. Cell surface GRP78 promotes tumor growth through stabilization of checkpoint protein PD-L1, oncofetal proteins ROR1, and Cripto (tumor promotion). CBT300 and CBT200 remove the surface GRP78, eliminating its stabilization of PD-L1, ROR1, and Cripto which then induces tumor apoptosis and reducing drug and immune resistance (tumor apoptosis). GBM, glioblastoma multiforme; GRP78, glucose-regulated protein 78; PD-L1, programed death-ligand 1; ROR1, receptor tyrosine kinase–like orphan receptor-1; CBT, Creative BioTherapeutics.

A recent study demonstrated that ROR1 is an upstream regulator of the expression of ABCB1 (P-glycoprotein) and ABCC1 (MDR-1) drug efflux pumps (45, 46). Doxorubicin is a known substrate for the ABCB1 drug efflux pump. Since we have shown that extracellular GRP78 can upregulate and stabilize surface ROR1, we wanted to determine whether extracellular GRP78 could increase drug resistance by decreasing internalized doxorubicin and whether CBT300 could reverse this drug resistance. Using the GBM cell line U87-MG, with extracellular GRP78 (5 μg/ml), doxorubicin (2 μM), and CBT300 (200 nM), the amount of doxorubicin internalized was measured by flow cytometry and immunohistochemistry (IHC) due to the natural red fluorescence of doxorubicin. In Figure 6, *A* and *B* extracellular GRP78, when bound to cells, significantly reduced doxorubicin internalization by almost 50%. However, when CBT300 was added to the cells, the number of cells with internalized doxorubicin dramatically increased by over 250%. IHC analysis was performed to validate the results. Doxorubicin (red) internalization was significantly enhanced by approximately 250% (*p* < 0.0001) when CBT300 was added to U87 cells (Fig. 6, *A* and *B*). These data also show that surface-bound GRP78 was significantly reduced by 98% (*p* < 0.0001) when CBT300 was added.

### GRP78 inhibitor, K5, dosed systemically leads to complete orthotopic brain tumor regressions and improved overall survival in mice

**Figure 7:**
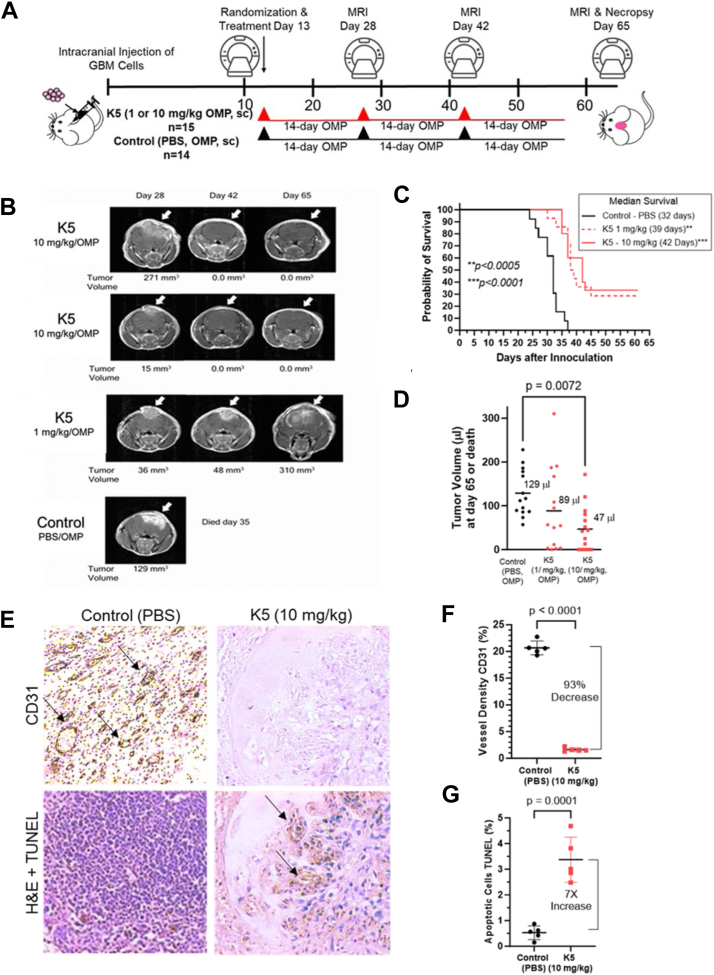
**Antiangiogenesis and GRP78 inhibitor K5 (kringle 5), regresses GBM tumors and significantly extends survival with durable complete regressions in an orthotopic glioma murine model.***A,* experimental setup of intracranial stereotactic injections with D54 adult GBM cells in the brains of nude mice and subsequent treatment with K5(3 X14-day osmotic mini pumps) and MRI analysis on days 28, 42, and 65 after tumor cell inoculation. *B,* MRI analysis of orthotopic adult GBM D54 tumors treated with and without K5. *C,* Kaplan–Meier curves showing survival of K5-treated mice *versus* controls. *D,* GBM D54 tumor volume at day 65 or at death as determined by MRI analysis. *E–G,* immunohistochemical analysis of GBM D54 tumor tissues were analyzed for vessel density (CD31) and apoptotic cells (TUNEL) at day 65. Five tissue samples were stained from each group and the average with SD were plotted. Survival curves in (*C*) were compared using log-rank test. Statistical analysis in (*D*) was performed using two-way ANOVA with *post hoc* Tukey’s test. Statistical analysis in (*E*) and (*F*) was performed using one-way ANOVA with *post hoc* Dunnett’s test. CCK-8, Cell Counting Kit-8; GBM, glioblastoma multiforme; GRP78, glucose-regulated protein 78.

To determine whether surface-bound GRP78 inhibition by systemic dosing with a known inhibitor, K5, leads to glioma tumor regression and increased survival *in vivo*, we used an orthotopic glioma nude mouse model implanted with human patient-derived adult D54 GBM cells. The cells were stereotactically implanted into the brains of the nude mice. After 13 days, the mice were randomized into three groups: control (PBS, sc), K5 (10 mg/kg, sc), and K5 (1 mg/kg, sc). Because of the poor pharmacokinetics of K5 (half-life = 20 min in mice), K5 was delivered by osmotic minipumps (OMP, 14 days, 0.25 ml/h) that were changed every 14 days for a total of three OMPs used for each group (Fig. 7*A*). Minipumps were implanted into the sc backs of nude mice. Gadolinium-enhanced mouse MRI brain sections were obtained on days 28, 42, and 65. Mouse brains were collected and analyzed after death or day 65. In Figure 7*B*, the images show the gadolinium-enhanced mouse MRI brain sections on days 28, 42, and 65 for two mice treated with K5 at 10 mg/kg/day, 1 mouse treated with K5 at 1 mg/kg/day, and one control mouse treated with PBS, OMPs, or sc. In the K5 10 mg/kg/day treatment group, more than 30% (5/15 mice) of the mice had complete tumor regression, as determined by the comparison of MRIs from day 28 with those from days 42 and 65 (Fig. 7, *B*–*D*). White arrows depict the GBM tumor location. MRI analysis on day 65 of sequential 1.5-mm sections of complete brains showed no detectable tumors in these mice*.* In comparison, durable regression of the tumor mass was not observed in any of the control mice or in any mouse administered a lower dose of K5 (1 mg/kg/day, sc). Although the lower dose of K5 significantly extended survival time by slowing tumor growth by approximately 40 to 45%, no tumor regression was observed (Fig. 7, *C* and *D*). The greater than 30% increase in median survival was significant (*p* < 0.0005) in all K5-treated groups compared to that in the control group (Fig. 7*C*). The final tumor volume was also measured using MRI either at death or on day 65. A significant 64% reduction in brain tumor mass was observed in K5 (10 mg/kg/day)-treated mice (*p* < 0.0072) (Fig. 7*D*).

Our previous studies demonstrated that K5 is a potent angiogenesis inhibitor (12, 13). To test whether D54 brain tumors have decreased vessel density when treated with K5, immunohistochemical (IHC) analysis of brain D54 GBM tissue was stained for CD31 (endothelial cells). With K5 treatment at 10 mg/kg, IHC analysis of CD31 stained tissue sections showed a 93% decrease in vessel density (*p* < 0.0001) compared to PBS control GBM tissue sections (Fig. 7, *E* and *F*). As we have also shown that K5 treatment of GBM tumor cells induces apoptosis (12, 13), we stained GBM tumor sections with TUNEL staining to determine the increase in apoptosis (Fig. 7, *E* and *G*). In K5-treated GBM tumor tissues, the number of apoptotic tumor/endothelial cells increased seven-fold (*p* = 0.0001).

### CBT300 (Kr1Fc) dosed systemically by sc injection leads to patient-derived xenograft (CTG-2687) glioblastoma tumor regressions and improved survival in mice

**Figure 8:**
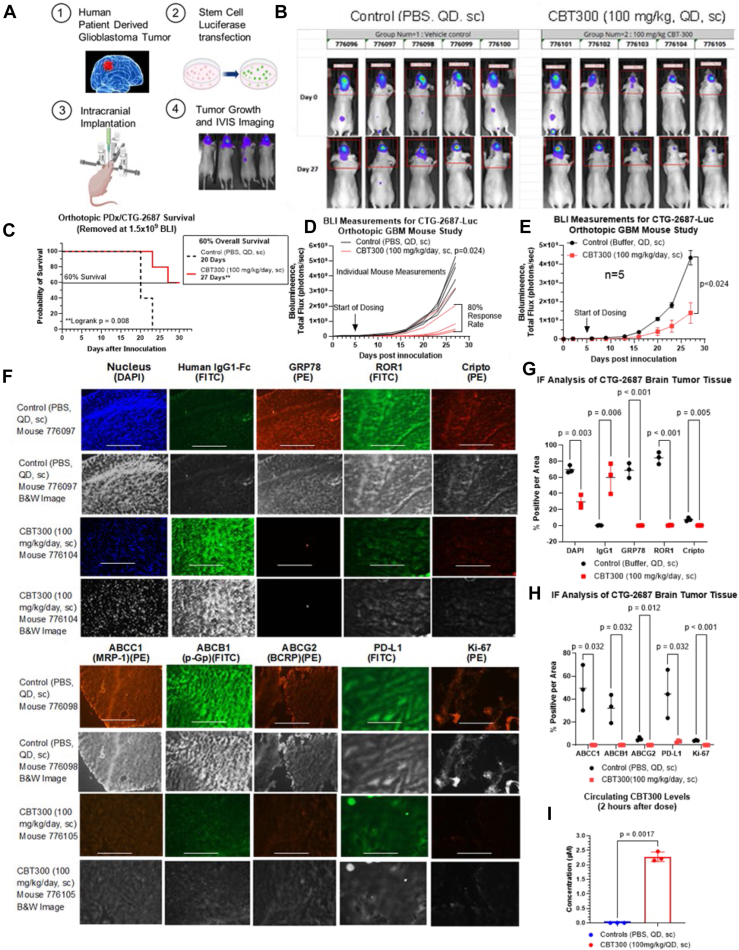
**GRP78 inhibition by systemic dosing of CBT300 (Kr1Fc) improves survival in preclinical models of glioblastoma cancer.***A,* experimental setup of stereotactic intracranial injection of adult PDx CTG-2687-Luc cells in the brains of nude mice and subsequent treatment with CBT300 (Kr1Fc). *B,* tumor bioluminescence of treated mice at days 0 and 27 of Control and CBT300-treated mice. *C,* Kaplan–Meier survival curve showing a significant 60% survival of CBT300 (Kr1Fc)-treated mice *versus* controls upon intracranial injection of PDx GBM cells. *D,* individual mice tumor volume as determined by bioluminescence. Control treated mice in *black lines*. CBT300-treated mice in *red lines. E,* average tumor volume as determined by bioluminescence for the CBT300 and Control groups up to day 27. N = 5 mice per group. *F,* immunofluorescence staining of CTG-2687 tumors for CBT300 and Control treatments. The scale bar represents 400 μm (10×). *G,* statical analysis of Control and CBT300-treated PDx GBM tumors for tumor cell number (4′,6-diamidino-2-phenylindole), CBT300 (IgG1), cell surface GRP78, ROR1, and Cripto expression as determined by immunofluorescence. Three independent mice GBM tumor tissues were measured. *H,* statical analysis of Control and CBT300-treated PDx GBM tumors for tumor expression of ABCC1, ABCB1, AGCG2, PD-L1, and Ki-67. Three independent mice GBM tumor tissues were measured. *I,* circulation concentrations of CBT300 in CBT300 treated and Control mice blood on day 27 2 h after last dose. Statistical analysis was performed using Kaplan–Meier survival curves in (C) and (D-H) were compared with an unpaired *t* test analysis using a two-stage step-up method by Benjamini, Krieger, and Yekutieli. GBM, glioblastoma multiforme; GRP78, glucose-regulated protein 78; PD-L1, programed death-ligand 1; ROR1, receptor tyrosine kinase–like orphan receptor-1; DIPG, diffuse intrinsic pontine glioma; PDx, patient-derived xenograft; CBT, Creative BioTherapeutics.

Because of the poor half-life of K5 and the impracticality of using minipumps in human patients, we developed CBT300 (Kr1Fc, 75 kDa) that has a half-life of a sc injected CBT300 of 10 to 11 days compared with 20 min for K5 in mice. Because of the improved half-life, increased affinity for GRP78 and its increased efficacy against glioma tumor cells *in vitro*, we tested CBT300 in a patient-derived xenograft (PDx) orthotopic athymic mouse tumor study. This study was conducted by Champions Oncology with human PDx glioblastoma (CTG-2687) luciferase stem cells. These GBM cells, once removed from the tissue, had been grown in neural basil stem cell media (with no fetal bovine serum [FBS], only 15 ng/ml epithelial growth factor [EGF], and 15 ng/ml basic fibroblast growth factor [bFGF]) and labeled with Luciferase in less than three cell splits. Adult PDx GBM (CTG-2687-Luc) stem cells were stereotactically implanted into the brains of athymic immune compromised mice. After 5 days, when tumors were viable by IVIS imaging, the mice were dosed with either CBT300 at 100 mg/kg/day, sc, or PBS by sc injections daily between the shoulder blades (Fig. 8*A*). Figure 8, *B*–*D* show that sc administration of CBT300 at 100 mg/kg/day reduced the mean brain tumor volume by 75% (*p* = 0.024). Complete responses, as determined by MRI and histological analysis, were observed in 60% (3/5) of mice with established CTG-2687 GBM tumors. At the end of the study, day 30, 60% (*p* = 0.008) of the CBT300 (100 mg/kg/day)-treated mice were still alive compared with 0% of the control mice resulting in a 7-days (*p* = 0.008) increase in the 60% survival rate with CBT300 treatment. IF and histopathological analysis of the mice brains from the control and the CBT300-treated group showed that several mice had no abnormalities or tumor cells detected in the brain tissue whereas the control group showed extensive neoplastic involvement; and the tumor(s) not well circumscribed; highly pleomorphic; numerous mitotic figures; and scattered foci of necrosis. (Fig. 8*E*).

### CBT300 crossed the BTB, circulates at about 2.2 μM and reduces cell surface expression of GRP78, ROR1, Cripto, MRP-1, MDR-1, and BCRP on PDx brain tumor cells

To determine if CBT300 bound to the GBM tumor cells in the mouse brain, we stained the brain tumor tissues for human IgG1 Fc domain from CBT300. In Figure 8, *E* and *F*, we show that about 60% (*p* = 0.006) of the cells had CBT300 bound (positive for human IgG1 domain). Since GRP78 and ROR1 have been linked to checkpoint proteins and drug efflux pump expression on tumor cells, we wanted to determine how CBT300’s inhibition of GRP78 affected these proteins. From Figure 8, *E*–*G*, we can show CBT300 reduces GRP78 surface expression by more than 99% (*p* = 0.0005). This reduction in surface expressed GRP78 leads to a significant decrease in expression of ROR1 (>99%, *p* < 0.0002), PD-L1 (93%, *p* = 0.032), Cripto (98%, *p* = 0.005), drug pumps: multidrug-resistant protein-1-ABCC1 (95%, *p* = 0.037), p-Glycoprotein-ABCB1 (>99%, *p* = 0.032), and breast cancer resistance protein-ABCG2 (95%, *p* = 0.013). In this study, CBT300 dosing showed no overt toxicity or mice having >20% weight loss, physical distress, or treatment related death. The scale bar is 400 nm (10×). Finally, we have measured the circulating concentrations of CBT300. Our results (Fig. 8*H*) show that CBT300 at 100 mg/kg/day is absorbed through the sc injection site and circulates at about 2.2 μM 2 h after a CBT300 dose.

## Discussion

Even with the best available treatments for patients with brain cancer, recurrence rates are nearly 100%, and therapeutic options at the time of relapse are extremely limited (1, 2, 3, 4, 5, 6, 7, 8). Recent studies have shown that the TME can induce drug resistance and cancer stem cell expansion, leading to recurrence (9, 10, 11, 12, 13, 14). How this TME can induce relapse and drug resistance is not well understood. Nevertheless, several studies have shown that the survival factor GRP78 is upregulated and can induce drug resistance in GBM tumors. Although it has been shown that cytoplasmic GRP78 is important for glioma growth and radioresistance, our focus was to demonstrate that surface-bound GRP78 could induce chemoresistance, immune resistance, and GSC expansion. Thus, the inhibition of surface-bound GRP78 could cause tumor regression, reduced drug resistance, lessened immune evasion, and decreased stem cell expansion.

Our results demonstrated that extracellular GRP78, at a concentration that has been shown to circulate in patients with several tumor types and in the cerebral spinal fluid of brain tumor patients (30, 31, 32, 33), can induce chemotherapy drug resistance by 5- to 20-fold in adult and pediatric GBM tumor cells. To further understand how extracellular GRP78 binding to GBM cell surfaces induces drug resistance, we isolated the cell surface proteins that bind to extracellular GRP78. The GRP78 receptor pull-down method ensured that only cell surface proteins that bind to extracellular and not intracellular GRP78 were isolated. Remarkably, we pulled down and identified two known cell surface GRP78-binding proteins, Cripto-1, PD-L1, and one previously unknown cell surface GRP78-binding protein ROR-1 (36, 37, 38). ROR1 is an oncofetal protein, which has been shown to be essential in stem cell formation, proliferation, metastasis, epithelial-to-mesenchymal transition, and drug resistance in many types of tumor cells including GBM cells (45, 46).

Our flow cytometry data demonstrated that ROR1 was highly expressed in pediatric SF9402 GBM cells and adult U87 GBM cells (43–62% of the cells). In addition, IHC analysis of adult U87 GBM cells showed that GRP78 and ROR1 colocalized on the surface of GBM cells. As it has been reported that CD44, a stem cell marker, binds to GRP78, we compared the binding affinities of PD-L1, Cripto-1, ROR1, and CD44 to GRP78. The ECDs of these proteins binding to extracellular GRP78 clearly show that PD-L1, ROR1, and Cripto-1 bind with affinity (Kd) for GRP78 in the nanomolar range and are 20 to 100× more potent than CD44.

Next, using IHC analysis of normal human brain and brain tumor tissues, we determined that ROR1, Cripto-1, and PD-L1 colocalized with GRP78 in adult GBM tumor tissues compared with normal brain tissues. We also showed that GRP78, ROR1, PD-L1, and Cripto-1 were highly expressed in late-stage GBM brain tissues compared to normal brain tissues. This confirms our pull-down and *in vitro* cell-based experiments, where GRP78 binds to ROR1, Cripto-1, and PD-L1 in GBM cells and tumor tissues.

Since the binding of extracellular GRP78 to ROR1 has not been previously reported, we wanted to determine how ROR1 and extracellular GRP78 bind. After examination ROR1’s ECDs, we identified that ROR1 has a kringle domain that is over 50% identical to our previously published GRP78 inhibitor, Kringle 5 (K5), from human plasminogen (12, 13). Using *in silico* docking programs, we identified that the ROR1 kringle domain binds to GRP78 with predicted energy and interactions like those of K5. The Kr1 and K5 kringle domains were 55% identical with greater than 90% homology. It was not surprising that we found the only ECD of ROR1 bound to GRP78 was the kringle domain (Kr1). Since we know that K5 has a poor half-life, we created two innovative GRP78 inhibitor fusion proteins containing either the K5 domain or the ROR1 kringle domain fused to an IgG1 heavy chain. Comparing these two therapies in Table 2, we show that between K5 (CBT500), K5Fc (CBT100), and Kr1Fc (CBT300), CBT300 has a significantly increased binding affinity for GRP78 that is 867× more potent than that of CBT500(K5) and 100× more potent than CBT100 (K5Fc). CBT300 and CBT100 are highly expressed in CHO cells, and using a simple purification method with protein A, CBT300 can be purified to over 99% purity, whereas CBT100 shows larger molecular weight aggregations. These data indicate that CBT300 is a promising lead candidate. Protein A and the secondary benzamidine column binding of CBT300 ensure that the IgG1 tail and kringle domain are folded properly (42).

**Table 2:** Characteristics of GRP78 inhibitors

Common name	GRP78 inhibitors			
	CBT100	CBT200	CBT300	CBT500
	K5Fc	K5PEG	Kr1Fc	K5
Kd binding to GRP78	30 nM	65 nM	**0.3 nM**	260 nM
IC50 2D Proliferation assay (U87)	54.8 nM	116.8 nM	**2.5 nM**	494 nM
IC50 2D proliferation assay (U118)	11.7 nM	33.2 nM	**1.3 nM**	49.4 nM
Half-life (mice–sc)	8 days	2 h	**11 days**	0.2 h
Aggregation (expiCHO)	20%	10%	**<1%**	<5%
Yield from expiCHO (mg/ml)	1	1.5	2	**3**
Purity after protein A or benzamidine column	80%	90%	**99%**	96%

CBT, Creative BioTherapeutics; GRP78, glucose-regulated protein 78; sc, subcutaneous.

Bold values indicate the best results.

CBT300 eliminates cell surface GRP78, which blocks stabilization of ROR1, PD-L1, and Cripto-1. The half-life of CBT300 in mice was significantly improved compared with that of K5 (11 days *versus* 20 min). Our data also show that CBT300 potently (nM) inhibits the proliferation of adult and pediatric glioma cell lines, with IC50 values at low nanomolar concentrations in 2D cell assays. In addition, studies using adult 827 GBM stem cells grown in modified stem cell media and attached to laminin showed a CBT300 dose response and a significant 75% reduction in cell viability. The addition of extracellular GRP78 to adult GBM and pediatric DIPG cells directly increased their resistance to doxorubicin. CBT300 reverses chemoresistance by blocking GRP78 binding and significantly reducing ROR1, Cripto, and PD-L1 expression. To prove this statement, our data in Figures 5 and 6 show that extracellular GRP78 added to pediatric DMG and adult GBM cells increased ROR1 (40%), Cripto-1 (188%), and PD-L1 (55%) expression. However, addition of CBT300 after addition of extracellular GRP78 completely inhibited GRP78, ROR1, Cripto-1, and PD-L1 surface expression.

Recent reports demonstrated that glioma cells cultured in 3D neurospheres were 5 to 8 times more resistant to chemotherapy than glioma cells attached in 2D assays (43, 44). Glioma cells cultured in 3D neurospheres were more stem-like with higher expression of drug efflux pumps and were more representative of clinical High-Grade Gliomas (43, 44). Because of this, we used 3D glioma cultures to determine if CBT300’s inhibition of extracellular and surface GRP78 could increase doxorubicin internalization and regression of neurospheres. Our data demonstrate for the first time that the inhibition of surface-bound GRP78 by CBT300 can reduce 3D tumor growth in adult and pediatric gliomas. The data showed that the combination of CBT300 and doxorubicin resulted in synergistic inhibition of chemoresistance, leading to a significant 50% to 100% regression in neurosphere size. This study included 827 patient-derived stem cells grown in 3D culture.

Finally, our data also showed, using two different methods, that CBT300’s reduction of surface GRP78, ROR1, PD-L1, and Cripto-1 significantly increased the internalization of chemotherapy (doxorubicin) in glioma tumor cells. Since ROR1 and Cripto-1 expression has been shown to increase the expression of ABCC1 and ABCB1, we propose that the GRP78/ROR1/Cripto-1/PD-L1 axis is the mechanism of action of extracellular GRP78 in increasing chemoresistance, immune evasion, and cancer stem cell expansion. We also showed that CBT300 removes surface GRP78 by eliminating chemoresistance, immune suppression, and cancer stem cell expansion. Our hypothesis (Fig. 6*C*) is that extracellular GRP78 in the TME binds to ROR1, Cripto, and PD-L1 on Glioma cells to regulate and stabilize their expression, and that CBT300 removes GRP78, reversing this protumorigenic phenotype.

Inhibition of extracellular and cell surface GRP78 increases *in vivo* survival and decreases tumor volume in preclinical models of GBM. K5 dosed with sc OMPs or CBT300 (Kr1Fc) dosed with sc injections significantly increased survival and reduced GBM orthotopic tumor volume, with no observable adverse effects or weight loss in preclinical *in vivo* models. Our *in vitro* data showed that surface GRP78 inhibition resulted in the almost complete elimination of ROR1, PD-L1, and Cripto-1. As the *in vivo* GBM preclinical models were performed in immunocompromised nude mice, we concluded that the decrease in tumor PD-L1 expression did not have a significant effect on tumor regression or survival. However, the decrease in ROR1, Cripto-1, and surface GRP78 displayed significant reductions in the viability and regression of glioma spheroids in multiple adult and pediatric brain tumors. Even in difficult-to-inhibit patient-derived adult GBM stem cells, surface GRP78 inhibition with CBT300 resulted in decreased cell viability and significant spheroid regression. These results strongly suggest that K5 and CBT300 cross the blood-tumor barrier in both D54 and PDx CTG-2687 adult GBM tumors.

Inhibition of extracellular and surface GRP78 with inhibitors, such as CBT300, or K5, eliminates surface-bound GRP78, ROR1, Cripto, and PD-L1, leading to chemo sensitization, reduced immune evasion, reversal of GSC formation, and increased tumor regression. We cannot exclude that some surface GRP78 may also be directly derived from the ER. However, GRP78 inhibitors that do not enter the cell and directly target surface GRP78 expression are preferable and advantageous over current GRP78 inhibitor therapies, because of the ubiquitous expression of intracellular GRP78 in normal cells. Our lead GRP78 inhibitor, CBT300 was chosen due to its excellent binding affinity to GRP78, long half-life, and lack of aggregation that is problematic with CBT100 (K5Fc) (Table 2). Further studies are needed to provide a more comprehensive understanding of how surface GRP78 induces drug resistance and tumor cell survival in multiple tumor types. In conclusion, our study provides preclinical and translational evidence that the inhibition of surface-bound GRP78, along with low doses of chemotherapy, could lead to a paradigm shift in the treatment of adult and pediatric glioma patients.

## Experimental procedures

### Reagents and antibodies

Proteins were labeled with Lightning-Link conjugation kits with HRP (Abcam, Cat# ab102890), PE (Abcam, Cat# ab102918), FITC (Abcam, Cat# ab188285), and biotin (Abcam, Cat# ab201795) according to the manufacturer’s instructions. Doxorubicin hydrochloride (Sigma-Aldrich Cat# 44583), GRP78 (StressMarq Cat# His-SPR-107C, SPR-119C, ATPase active domain containing no ADP or ATP), ROR1 ECD (ACRO Biosystems Cat# RO1-522y, Gln30- Glu403), ROR1 Ig-like domain (ACRO Biosystems Cat# RO1-H5221, Glu39–Gly151), ROR1 kringle domain (ACRO Biosystems Cat# RO1-H5223, Asn308–Asp 395), ROR1 Frizzled domain (ACRO Biosystems Cat# RO1-H5222, Glu165–Asp305), Cripto (Human TDGF1,fc ACRO Biosystems Cat# CRO-H5253), CD44 ECD (Sino Biological Cat# P16070-1, Met1-Pro220) were used per manufacturer’s instructions. To detect live cells, we used CCK-8 (APExBIO Cat# K1018,). The following primary antibodies were used for IF are listed in Table S1. Other reagents and immunofluorescent stain used was DAPI-Prolong Gold (Thermo Fisher Scientific, Cat# p36931). PBS pH 7.2 (Thermo Fisher Scientific, Cat# 10010023) was used for all cell washes dilutions.

### Cell lines

The primary pediatric GBM cell lines, SF9402 and SF9427, and the DIPG cell line, SF8628, were generously gifted by Rintaro Hashizume, MD, PhD, Northwestern University. The DIPG cell lines DIPG13, DIPG24, DIPG38, DIPG48, and DIPG50 were generously gifted by Michelle Monje, MD, PhD, at Stanford University. The 827 patient-derived GSCs were a generous gift from Dr Jeongwu Lee at the Cleveland Clinic and used solely by Dr Candece Gladson’s lab at the Cleveland Clinic. The adult and pediatric GBM and DIPG cells lines from Northwestern and Stanford were authenticated by short tandem repeat profiling and flow cytometry analysis. Two adult GBM cell lines, U87 (RRID:CVCL_3428) and U118, were purchased from American Type Tissue Culture (ATCC-Maryland). Our cell cultures are continuously monitored for doubling times and morphology, and they are tested for contamination from *mycoplasma* and bacteria using standard detection kits. Each culture is passaged less than 20 times.

### 2D proliferation assay

Cell proliferation was determined using the 3-(4,5-dimethylthiazol-2-yl)-2,5-diphenyltetrazolium bromide cell viability assay. Briefly, 5000 HGG (U118, U87, DIPG13, DIPG38, DIPG50, SF9402, SF9427, and SF8628) cells per well in Dulbecco’s modified Eagle’s medium (DMEM containing 10% FBS) were incubated in a 96-well plate (CELLTREAT, #229195) with and without GRP78 (5 μg/ml) overnight at 37 °C and 5% CO_2_. CBT300 was added at various concentrations (435.6 μg/ml–5.58 ng/ml) and incubated for 5 days. The number of live cells was then determined using a CCK-8 (Dojindo), and absorbance was measured at 450 nm. For the U118 and DIPG13 cell proliferation assays with doxorubicin and GRP78 (5 μg/ml), 5000 cells per well were incubated in full medium (DMEM containing 10% FBS) in 96-well plates overnight at 37 °C and 5% CO_2_. Decreasing concentrations of doxorubicin from 50 μg/ml to 0.05 ng/ml were added and incubated for 72 h. After incubation, CCK-8 was added to each well to determine the number of viable cells by measuring absorbance at 450 nm. Four replicate wells were used for each concentration point.

Adult 827 GSCs were grown in neurobasal medium (Thermo Fisher Scientific, 21,103,049) with 20 ng/ml EGF (Thermo Fisher Scientific, PHG0311) and 20 ng/ml bFGF (Thermo Fisher Scientific, AF-100-18B-50UG). The cells were grown on laminin-coated plates and flasks. For 2D proliferation assays, cell media were removed and attached cells were removed with TrypLE (Thermo Fisher Scientific, 12563011), counted using trypan blue, and new neurobasal media with EGF and bFGF was added to the cells. A total of 5000 cells per well were added to laminin-coated 12-well plates and incubated overnight for cell attachment. The next day, CBT300 was added to the wells at 550 nM and 275 nM, along with the negative control PBS and the positive control staurosporine (100 nM) (Cell Signaling, 99535) to the wells. The cells were then incubated at 37 °C and 5% CO_2_ for 7 days, and the number of dead cells was measured using trypan blue. Each data point represents data from individual microscope fields from three wells of a 12-well plate.

### 3D neurosphere regression assay

Neurosphere regression assays were used to compare tumor regression between control, GRP78, CBT300, and doxorubicin (Sigma-Aldrich, D1515)-treated HGG neurospheres. Glioma cells (DIPG13, DIPG24, DIPG38, DIPG48, DIPG50, and U118) were cultured as spheres in nonbinding 96-well plates (Nunclon Sphera 96U or 96F; Thermo Fisher Scientific). The neurospheres were then removed and broken apart to prepare single-cell suspensions using TrypLE (Thermo Fisher Scientific, 12563011). Single cells were counted *via* flow cytometry using ViaCount (Luminex, 4000-0040). The resuspended cells were then diluted in equal amounts with spent media and fresh tumor stem cell media (TSM) to achieve a density of 10,000 cells/ml. One hundred microliters of suspended cells were added to each well with and without GRP78 at 5 μg/ml in a new 96-well plate (Nunclon Sphera 96U or 96F, Thermo Fisher Scientific) low-binding wells and centrifuged at 300×*g* for 5 min before incubating at 37 °C and 5% CO_2_. After 48 h of incubation for spheres to form, CBT300 (550 nM, 275 nM), temozolomide (5 mM) (Sigma-Aldrich, T2577), and doxorubicin (36.7 μM) at various combinations were added to wells. The neurospheres were then incubated for at least an additional 72 h. “Day 0” denotes the day that treatments were added to the established neurospheres. Microscopic images were taken on various days for each neurosphere, from day 0 to day 27. To prepare the TSM base, 250 ml of Neurobasal-A (Thermo Fisher Scientific, 10888022) and 250 ml of DMEM: Nutrient Mixture F12 (DMEM/F12) (Thermo Fisher Scientific, 21041025) were mixed with 5 ml of 100× antibiotic-antimycotic (Thermo Fisher Scientific, 15240062), 5 ml 200 mM L-alanyl-L-glutamine dipeptide (GlutaMAX-A, Thermo Fisher Scientific, 35,050,061), 5 ml Hepes buffer (Thermo Fisher Scientific, 15630080), 5 ml 100 mM sodium pyruvate (Thermo Fisher Scientific, 11360070), and 5 ml 100× MEM nonessential amino acids (Thermo Fisher Scientific, 11140035).

To prepare complete TSM with growth factors, the following supplements were added to the TSM base: 50× B27 Supplement Minus Vitamin A (1:50) (Thermo Fisher Scientific, 12587010), human epidermal growth factor (20 ng/ml), human fibroblast growth factor (20 ng/ml), human platelet-derived growth factor AA (10 ng/ml) (Thermo Fisher Scientific 100-13A-10ug), human platelet-derived growth factor BB (10 ng/ml) (Thermo Fisher Scientific, 100-14B-10UG), and heparin solution (2 μg/ml) (Stem Cell technologies, 07980).

Neurosphere regression assays were also performed on 827 GSCs. The cells were grown in neurobasal medium (Thermo Fisher Scientific, 21103049) with 20 ng/ml EGF (Thermo Fisher Scientific, PHG0311) and 20 ng/ml bFGF (Thermo Fisher Scientific, AF-100-18B-50UG). Single cells were plated on uncoated 24-well plates and allowed to form spheres for 4 days prior to treatment with either 550 nM CBT300 or 550 nM control. The total number of spheres containing at least five cells was counted at 0, 3, 5, and 7 days after treatment in each well. The data are represented as the percentage of spheres remaining from the number of spheres counted on day 0 in triplicate.

### In vitro pull-down assay of SF9402 DIPG and U87 GBM cells

A. **Surface protein biotinylation**: SF9402 and U87 cells were used to pull down GRP78-His binding cell surface proteins. Two T75 cm^2^ flasks containing SF9402 and U87 HGG cells at >85% confluence were used. The medium was removed, the attached cells were washed with 10 ml PBS per flask, and the PBS was removed quickly. Using the Pierce Cell Surface Protein Biotinylation and Isolation Kit (Thermo Fisher Scientific, A44390), cell surface proteins were biotinylated by adding Sulfo-NHS-SS-Biotin to the flasks and incubating each flask for 10 min at room temperature per manufacturer’s instructions The Biotin labeling solution was then removed, and the cells were washed twice with 10 ml per flask of ice-cold Tris-buffered saline (TBS). The cells were scraped off with a cell scraper in 5 ml TBS per flask. Each type of scraped biotinylated cell to a 50 ml conical tube, one for SF9402 cells and one for U87 cells on ice. The flasks were washed with an additional 5 ml of TBS and added to 50 ml tubes for each cell type. Tubes containing biotinylated cells were centrifuged at 500*g* for 3 min at 4 °C. The supernatant was discarded from the cell pellet.
B. **Cell lysis**: Biotinylated cell pellets from SF9402 and U87 cells were lysed using lysis buffer (0.5 ml of lysis buffer mixed with protease inhibitors (Thermo Fisher Scientific, HALT Protease Inhibitor, 87786). The cell pellets were pipetted up and down to resuspend the cells. The cells were then transferred to a 1.5 ml microcentrifuge tube and incubated on ice for 30 min with occasional mixing. The tubes were then centrifuged at 15,000*g* for 5 min at 4 °C. The supernatant was then collected in a new 1.5 ml microcentrifuge tube.
C. **Cell surface biotinylated protein isolation**: After washing two microtube columns containing 250 μl of NeutrAvidin Agarose (included in the kit), the clarified biotinylated cell lysate supernatants were added to the capped column and incubated for 30 min at room temperature with end-over-end mixing. The columns were placed in new centrifuge tubes and centrifuged at 1,000*g* for 1 min. The flow-through was discarded. Five hundred μιλλιλιτερσ of wash buffer were added to the columns and mixed by inversion two to three times. The columns were then centrifuged again at 1000*g* for 1 min and the wash was discarded. The bound biotinylated cell surface proteins were eluted with 200 μl elution buffer and DTT. The capped columns were incubated at room temperature for 30 min with an end-to-end mixing. Surface-bound proteins were eluted from the columns by centrifugation for 2 min at 1000*g*. The eluates were then dialyzed using mini dialysis devices (3500 molecular weight cut-off [MWCO], Thermo Fisher Scientific, 88400) against PBS (pH 7.2) to replace DTT and the elution buffer.
D. **Binding of cell surface proteins to GRP78**: Surface proteins from SF9402 and U87 cells in 1.5 mml Eppendorf tubes were mixed with 1 μM GRP78-His and incubated with gentle mixing at 4 °C overnight. Cell surface proteins bound to GRP78-His were then added to 1 ml nickel-nitriloacetic acid (Ni-NTA) agarose (R90101, Thermo Fisher Scientific) gravity columns. The samples were gently mixed with an Ni-NTA column by rotation for 120 min. The column was then washed with three volumes of wash buffer (PBS with 20 mM imidazole, Ni-NTA-agarose kit, Thermo Fisher Scientific). The bound GRP78-His membrane proteins were eluted with elution buffer (PBS with 250 mM imidazole; Thermo Fisher Scientific). Samples were loaded into a SurePAGE 4 to 20% bis-tris gel (GenScript) and ran *via* electrophoresed using Mops buffer. Bound proteins were visualized with Coomassie staining, and protein bands were excised for mass spectrometry analysis at the Integrated Molecular Structure Education and Research Center in the Northwestern.

### IF of human brain tumor tissue arrays

IF of human brain tumor arrays was carried out using the protocol suggested by Cell Signaling Technology’s suggested protocol (www.cellsignal.com). Briefly, three human brain tumors and normal brain tissue microarrays (T174c; Biomax. us) were deparaffinized with xylene and washed with PBS. The slides were washed with 100%, 95%, and 70% ethanol for 5 min each. The slides were then dried overnight at room temperature. The slides were washed twice with PBS for 5 min and blocked with SuperBlock blocking buffer (37,515, Thermo Fisher Scientific) for 20 min at room temperature. Fluorescently FITC and PE primary antibodies (diluted 1:5000 in PBS) to GRP78, ROR1, GRP78, and Cripto, or GRP78 and PD-L1, were added to the slides and incubated overnight at 4 °C in the dark. The following day, slides were washed twice with PBS and air-dried at room temperature. Prolonged Gold with DAPI mounting solution was added to the dried slides, and a cover slip was carefully added to prevent bubbles. The slides were incubated overnight at room temperature in the dark, cleaned, and observed under an evolved vision optical system fluorescence microscope. Stained brain tumor images were compared with normal brain tissue images for the expression of GRP78, ROR1, PD-L1, and Cripto.

### IHC for brain tumor tissues

D54 human glioma tissue slides were deparaffinized with xylene as previously described. Sections were blocked with SuperBlock blocking buffer for 20 min at room temperature. The sections were then incubated with a Goat Anti-Human/Mouse/Rat CD31/PECAM-1 Antigen Affinity-purified Polyclonal Antibody (AF3628, R&D Systems) at 10 μg/ml was added to the sections overnight at 4 °C, followed by incubation with the Anti-Goat IgG VisUCyte HRP Polymer Antibody (VC004, R&D Systems) for 60 min at room temperature. The sections were washed 3 times for 10 min each with PBS. Hydrogen peroxide/DAB solution (34001; R&D Systems) was added to the sections for 4 to 5 min and washed with PBS. Tissue sections were counterstained with hematoxylin-eosin (ab245880, Abcam) for 30 s. The slides were then rinsed with water and mounted with ProLong Gold reagent under coverslips. The slides were dried overnight and visualized under an evolved vision optical system microscope. For TUNEL staining of glioma sections, a TUNEL assay kit (ab206386, Abcam) was used, according to the manufacturer’s instructions.

### Expression and purification of CBT300, CBT200, CBT100, and kringle domains

We developed novel GRP78 inhibitors based on the extracellular GRP78 binding kringle domains of ROR1 (Kr1) and plasminogen kringle domain (K5). The ROR1 (Kr1) kringle domain or plasminogen (K5) kringle domain was fused to the human IgG1 Fc domain and named CBT300 and CBT100, respectively. To express CBT300 or CBT100, the manufacturer’s instructions from Thermo Fisher Scientific on protein expression were followed. Briefly, the gene sequences of either the CBT100 (K5Fc) or Kr1Fc (CBT300) fusion proteins were inserted into the pTT5 plasmid and stably expressed in ExpiCHO cells (catalog A29133, Thermo Fisher Scientific) in serum-free ExpiCHO expression medium with yields of 1 to 2 mg/ml. The expressed fusion proteins were purified using a protein A plus Agarose Nab spin column (Catalog 89960, Thermo Fisher Scientific). Binding of Kr1Fc and K5Fc to a benzamidine-agarose column (catalog A8332, Sigma-Aldrich) as a secondary purification step confirmed that the kringle domains were folded properly (42). Purity measurements from densitometry analysis of SDS-PAGE gels and size exclusion chromatorgraphy-high pressure liquid chromatography (SEC-HPLC) analysis determined that CBT100 and CBT300 were at least 98% pure. CBT200 was produced from the kringle five fragment of plasminogen (12) conjugated to 40,000 Da monothio-polyethylene glycol (NHS PEG) (47, 48) (catalog 9699, Sigma-Aldrich), as previously described (47, 48).

### *In silico* protein docking analysis

The protein structures of GRP78 (UniProt P11021) and ROR1 (UniProt A2VCQ3) were retrieved from AlphaFold and UniProt. Protein–protein docking was executed using the docking tool ClusPro 2.0, in which GRP78 was used as the receptor and the K5 or ROR1 kringle domains as the ligands. Of the 10 possible docking models, the chosen model was selected based on the cluster size and lowest energy score, following developer recommendations. The docked structures were then analyzed using PyMOL (RRID: SCR_000305; www.pymol.org) with a cutoff set to 4.0 Å to obtain interacting residues between the receptor and the ligand. The interacting amino acids from the receptor match the known functional domain structure of GRP78. The docked structure interactions were observed using PBDsum, where the types of interactions were acquired (salt bridges, hydrogen bonds, and nonbonded contacts).

### Flow cytometry

Flow cytometry was used to determine cell surface proteins regulated by GRP78 and CBT300. Briefly, human high grade glioma (HGG) cells were incubated overnight in full media at 37 °C in 5% CO_2_ in 2 ml Eppendorf tubes with and without GRP78 (5 μg/ml). The cells were centrifuged, resuspended in PBS with and without CBT300 at a final concentration of (20 nM), and incubated for 6 h at 37 °C in 5% CO_2_ before washing three times in PBS. PE-labeled primary antibodies (0.5 mg/ml) were added to separate samples at a dilution of 1:1000 and incubated at room temperature in the dark for 30 min. The cells were then washed and resuspended in PBS. Samples were analyzed by flow cytometry using a Guava PCA system, and data were analyzed using FlowJo (RRID: SCR_008520; www.flowjo.com) software.

### D54 orthotopic glioma mouse model with K5 minipumps

All D54 animal experiments were conducted in accordance with the Institutional Animal Care and Use Committee guidelines and approved by the animal ethics committee. Using a stereotactic frame, one million D54 cells were implanted into the basal ganglia of male and female 6- to 8-week-old Nu/Nu CD1 mice. Only mice that displayed D54 tumors on day 12 by MRI analysis was included in the study. Tumor bearing mice were randomized on day 13 into three groups of n = 14 for Control and n = 15 each for the two K5 dose groups. OMPs containing K5 at 1 mg/kg/day or 10 mg/kg/day or vehicle (PBS) were implanted into the back flank of each animal on day 13. Mice were imaged on days 26, 42, and 65 to validate tumor growth, and the animals were maintained for the collection of survival data. These pumps can deliver K5 SC for 14 days. The OMPs were replaced every 14 days. MRI analysis was performed on a 4.7-T magnet using a solenoid volume radiofrequency probe 26, 42 and 65 days after inoculation. Mice were anesthetized and administered an i.p. injection of a gadolinium-based magnetic resonance contrast agent. A multi-slice, T1-weighted image was acquired from each mouse brain using a spin-echo imaging sequence. Neighboring transverse slices (1.5 mm) were acquired from the entire brain. The magnetic resonance signal within the tumor region was enhanced by the presence of a contrast agent because of the breakdown of the blood–brain barrier within the tumor. This signal enhancement allowed the differentiation of the tumor from normal brain tissue. The tumor volume was determined for each slice and summed across all slices to determine the total tumor volume. Mice that lost more than 20% body weight were imaged and removed from the study. In this study, the mouse attrition rate was 0% due to the low to no toxicity of the K5 treatments. Power analysis with a Wilcoxon–Mann–Whitney test for a two-sided unpaired sample power analysis using the ClinCalc.com website was used to determine the number of mice needed. The group size (n = 14) was powered to detect increases of at least 30% in overall survival between control and K5-treated groups, assuming a coefficient of variation equal to 1.5 (as suggested by projected/anticipated data) and using a two-sample *t* test for log normal data with 80% power and a significance level of 0.05.

### PDx CTG-2687 orthotopic glioma mouse models with systemic CBT300

The PDx CTG-2687 orthotopic glioma mouse models that were dosed with CBT300 were conducted in accordance with the Institutional Animal Care and Use Committee guidelines and approved by the animal ethics committee at Champions Oncology. Stereotactic injections of PDx CTG-2687-Luc GSCs were performed as previously described. In brief, on day 5, analysis was performed to include only mice with detectable tumors by IVIS imaging. Male and female 6- to 8-week-old Nu/Nu CD1 tumor-bearing mice were then randomized into groups of five mice each. Power analysis with a Wilcoxon–Mann–Whitney test for a two-sided unpaired sample power analysis using the ClinCalc.com website was used to determine the number of mice needed. The group size (n = 5) was powered to detect increases of at least 40% in overall survival between control and CBT300-treated groups, assuming a coefficient of variation equal to 1.5 (as suggested by projected/anticipated data) and using a two-sample *t* test for log normal data with 80% power and a significance level of 0.05. Samples of CBT300 (sample B) and a control (sample A) PBS sample were administered on day 5 after glioma cell inoculation. The dose of CBT300 given by sc injection was 100 mg/kg QD. Mice were removed from the study when weight loss reached >20%, or mice showed signs of distress or death. For the CTG-2687 GBM tumor study, tumor volumes were determined using IVIS imaging. Glioma tumors were measured for total bioluminescence flux which is representative of tumor volume. Creative BioTherapeutics (CBT) analyzed the tumor tissues for CBT300 (human IgG1), surface-bound GRP78, ROR1, PD-L1, Cripto, ABCB1, ABCG2, ABCC1, and Ki-67. CBT also measured circulating CBT300 in the plasma again by human IgG1 Fc analysis. The tissues were stained with mAbs that were validated by Western blot and IF analysis from various manufacturers and are listed in Table S1.

### Binding assays for GRP78 with ROR1 domains, Cripto, PD-L1, CD44

Binding assays for the ECDs of ROR1, Cripto, PD-L1, and CD44 were performed using ELISA with HRP/TMB reagents. ROR1 ECD, ROR1 kringle domain, ROR1 Frizzle domain, ROR1 IgG domain, Cripto, PD-L1 ECD, and CD44 ECD proteins were incubated at 1 μg/ml (100 μl) in PBS in triplicate wells of 96-well plates at 4 °C overnight. The plates were emptied and blocked with a blocking buffer (SuperBlock 37515, Thermo Fisher Scientific) for 30 min at room temperature. An ELISA-binding assay with HPR-labeled GRP78 was performed by adding various concentrations of GRP78-HRP to PBS and incubating for 2 h at room temperature. The plates were washed three times with 100 μl of PBS. Plates were then emptied and 100 μl of TMB 1-Step Ultra reagent (34028, Thermo Fisher Scientific) was added and incubated for 15 to 30 min until a dark blue color developed. The stop solution (100 ml, N600, Thermo Fisher Scientific) was added, and the plates were read on a spectrophotometer at 450 nm. Each data point was run in triplicate with one independent repeat. Student’s *t* test was calculated using *p* < 0.05 *t* test compared to GRP78-HRP binding alone. ∗*p* < 0.05 and ∗∗*p* < 0.001.

### Statistical analysis

All *in vitro* experiments were performed at least in triplicate. Data were summarized using descriptive statistics. Comparisons were made between the groups using the Wilcoxon rank-sum test or *t* test, whichever was appropriate for continuous variables. The changes from baseline to follow-up were compared using paired *t* tests. Survival time from the time of tumor cell injection was estimated using the Kaplan–Meier method, and differences in survival between groups were compared using the Wilcoxon test. GraphPad Prism 10 (RRID: SCR_002798; https://www.graphpad.com) software (GraphPad Software, Inc.), SAS 9.4, and R 3.3.2 were used for statistical analysis. Statistical significance was set at *p* < 0.05.

## Data availability

The data generated in this study are available upon request to the corresponding author.

## Supporting information

This article contains supporting information.

## Conflict of interest

D. J. D. and A. K. D. have ownership interests in Creative BioTherapeutics. DJD owns patents for CBT300 and CBT200. E. S., J. Z., M. T., and R. B. are employed by Creative BioTherapeutics. The other authors declare that they have no conflicts of interest with the contents of this article.
