# Supplementary material for: Inhibition of cell surface GRP78 on brain tumors reverses drug resistance and stops cancer stem cell expansion
Source: J Biol Chem. 2026 Jan 12;302(4):111146. doi: 10.1016/j.jbc.2026.111146 (PMC13052156; doi:10.1016/j.jbc.2026.111146)

Supporting Figure 1. CBT300 (Kr1Fc) Competes with K5 and other GRP78 inhibitions' binding to GRP78. Full length active GRP78 was added to 96 well plates to bind at 100 ng/well overnight at 4°C. The plates were then washed and blocked with superblock (ThermoFisher) for 60 minutes at room temperature. **A.)** 96 well plates were washed three times with PBS and different concentrations of either K5-biotin (C-terminal labeled) or ROR1 Kringle 1-biotin (C-terminal) was added to the plates and incubated at 4°C overnight. The plates were then washed and streptavidin-HRP was added to the wells at manufactures recommendation dilution of 1:10,000 and incubated for 30 min. The plates were then washed and TMB regent was added, and after about 2 hours equal volumes of stop solution (ThermoFisher) was added. Absorbance was recorded at 650 nm. Each point is an average of 3 wells. The Kd of K5-H3 has been reported at 0.81 nM so the K5 and Kr1 biotin reagents are binding like natural K5. **B.)** GRP78 was added to a 96 well plate as described above. 20 nM (at saturation point) of K5-biotin and various concentrations of GRP78 inhibitors, CBT300(Kr1Fc), CBT400(Kr1), CBT100(K5Fc) and unlabeled K5 (CBT500), were added and incubated at room temperature for two hours. The plates were then washed, and the analysis was completed as listed above. As can been observed from the data, the IC50 values for inhibition of K5 binding is CBT300(Kr1Fc)<CBT400(Kr1)<CBT100 (K5Fc) and finally CBT500(K5). **C.)** Time course analysis of SF9402 pediatric Glioblastoma cells with added extracellular GRP78 (5 ug/ml). Flow cytometry analysis (5000 cells) of SF9402 cells for cell surface bound GRP78. The data analysis shows a maximum increase in cell surface GRP78 at about 72 hours. The 96 hours flow cytometry shows an increase in Forward Scatter indicating some aggregation of cells. **D.)** After 72 hours of SF9402 cells incubated with GRP78, CBT300 was added at 100 nM. The binding of cell surface GRP78 was reversed by CBT300 within 24 hours. The 48 hours and 72 hours showed similar GRP78 inhibition as 24 hours.

Supporting Figure 1.

A. **Kingle Domain Binding to GRP78**

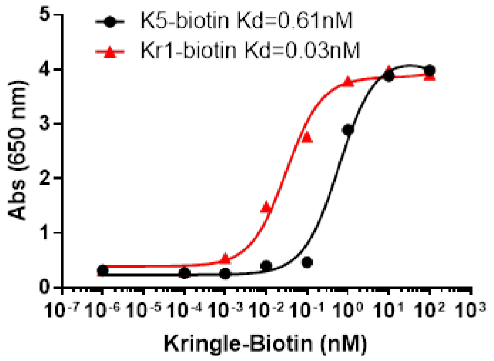

B. **Competition Binding Assay with 20 nM [illegible]5-Biotin on a GRP78 coated 96-well Plate**

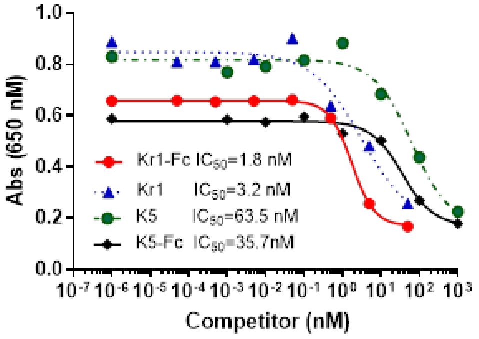

C.

Time Course: SF9402 Cells treated with 5 ug/ml GRP78 at Time 0 hours

Percent of SF9402 GBM Cells that are Positive for GRP78

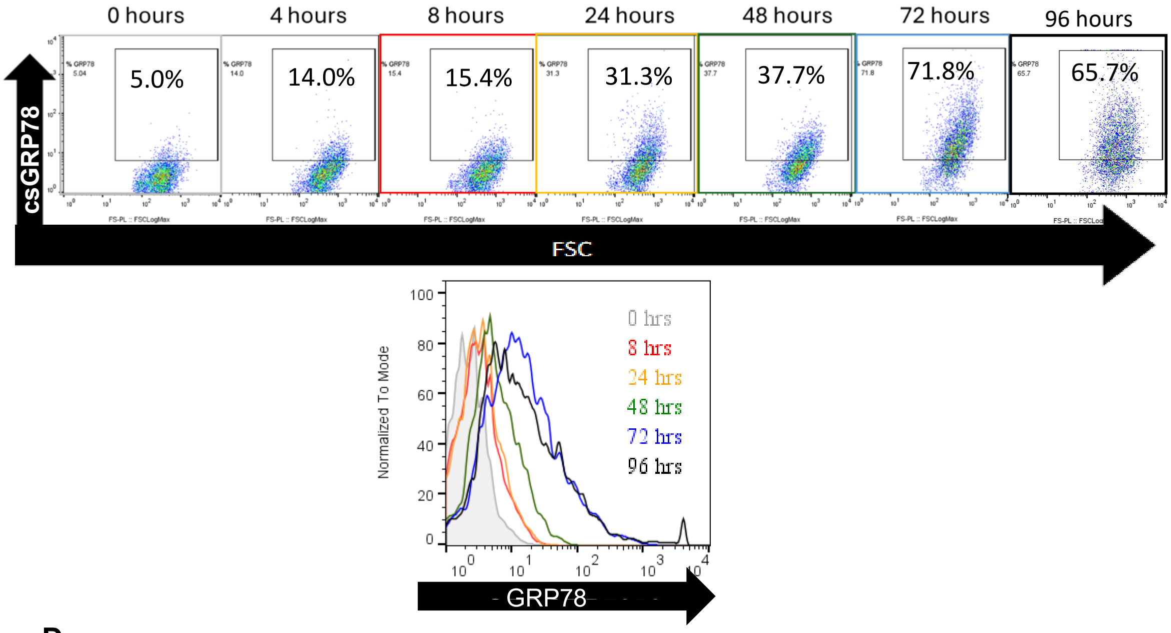

D.

CBT300 (100 nM)

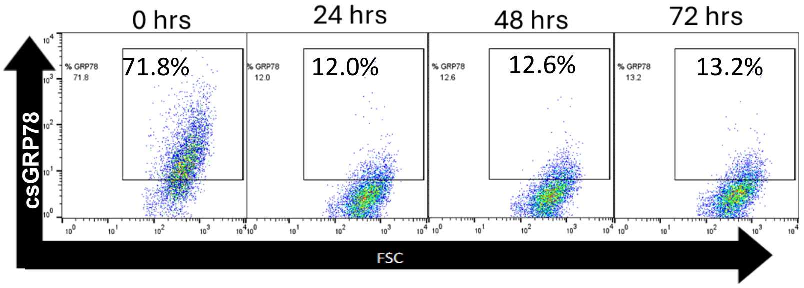

Supplement: Figure S1 [file mmc1.pdf]
