# Supplementary material for: Inhibition of cell surface GRP78 on brain tumors reverses drug resistance and stops cancer stem cell expansion
Source: J Biol Chem. 2026 Jan 12;302(4):111146. doi: 10.1016/j.jbc.2026.111146 (PMC13052156; doi:10.1016/j.jbc.2026.111146)
Supplement: Figure S2 [file mmc2.pdf]

Supporting Figure 2: CBT300 inhibits cell surface GRP78 leading to a decrease in cell surface expression of GRP78 binding proteins, ROR1 and PD-L1, on SF8628 and SF9427 glioma cells. A-B) SF8628, Diffuse Midline Glioma, H3 K27-altered cells and SF9427, High Grade Glioma H3-wild type, IDH-wild type cells were incubated at 37°C with 5 μg/ml extracellular GRP78 for 72 hours. The cells were washed with fresh media and various concentrations of CBT300 was added to the cells for 24 hours. Flow cytometry analysis of cell surface ROR1 and PD-L1 by flourescent antibody binding analysis. **C.)** Compiled analysis of CBT300 dose respone curve for ROR1 and PD-L1 expression on glioma cells. Each point is an average of 2000 glioma cells with one biological replicate.

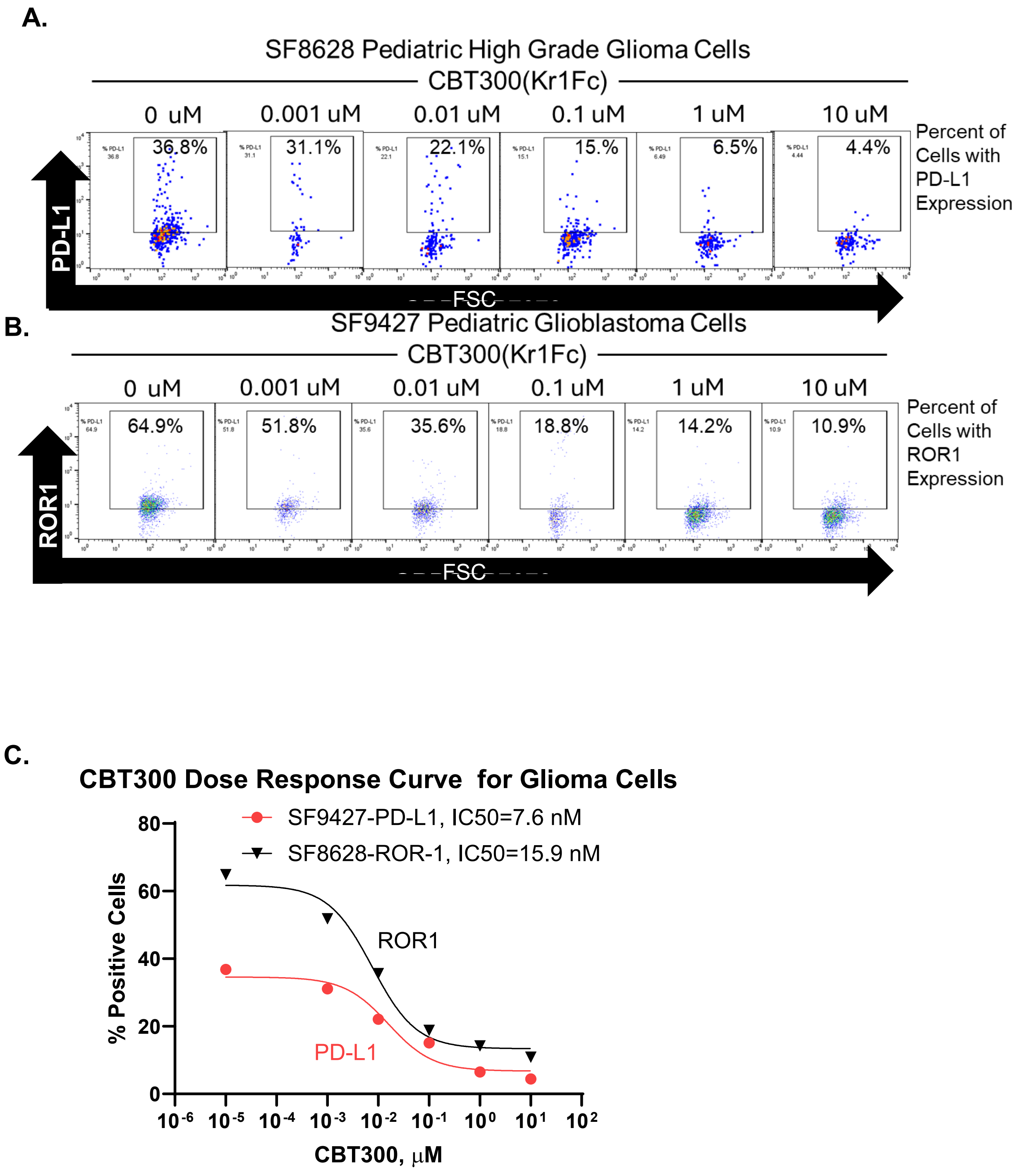
A.
SF8628 Pediatric High Grade Glioma Cells
CBT300(Kr1Fc)
0 uM
0.001 uM
0.01 uM
0.1 uM
1 uM
10 uM
36.8%
31.1%
22.1%
15.%
6.5%
4.4%
Percent of Cells with PD-L1 Expression
PD-L1
FSC
B.
SF9427 Pediatric Glioblastoma Cells
CBT300(Kr1Fc)
0 uM
0.001 uM
0.01 uM
0.1 uM
1 uM
10 uM
64.9%
51.8%
35.6%
18.8%
14.2%
10.9%
Percent of Cells with ROR1 Expression
ROR1
FSC
C.
CBT300 Dose Response Curve for Glioma Cells
SF9427-PD-L1, IC50=7.6 nM
SF8628-ROR-1, IC50=15.9 nM
ROR1
PD-L1
% Positive Cells
CBT300, µM
