# Supplementary material for: Inhibition of cell surface GRP78 on brain tumors reverses drug resistance and stops cancer stem cell expansion
Source: J Biol Chem. 2026 Jan 12;302(4):111146. doi: 10.1016/j.jbc.2026.111146 (PMC13052156; doi:10.1016/j.jbc.2026.111146)
Supplement: Table S1 [file mmc3.pdf]

Supporting Table 1. Antibodies Used for Analysis

Target	Antibody type	Label	Manufacturer	Cat #	Lot #	Species
GRP78	mAb	FITC	Santa Cruz	SC-13539	D2018	Mouse, rat, human
CD8	mAb	PE	Santa Cruz	SC-1177	F0413	Mouse, rat, human
ROR1	mAb	FITC	Miltenyi BioTech	130-104-647		human
Cripto	mAb	PE	R&D Systems	FAB2772P		human
CD44	mAb	PE	Invitrogen	12-0441-82		human, mouse
ABCG2	mAb	FITC	Santa Cruz	sc-377176	J0318	human
B7H3	mAb	PE	Biolegend	351003		human, monkey
PD-L1	mAb	FITC	Biolegend	393605		human
CD47	mAb	PE	Biolegend	323108		human, monkey
CD68	mAb	FITC	Biolegend	137006		Mouse
B7H4	mAb	PE	Biolegend	139405		Mouse
IgG1	mAb	FITC	Invitrogen	A55741	3118933	Human Only
Ki-67	mAb	PE	Biolegend	151210		Human, Mouse
ABCC1 (MRP-1)	mAb	PE	Santa Cruz Biotech	sc-53130		Human, Mouse, Rat
ABCG2 (BCRP)	mAb	PE	Santa Cruz Biotech	sc-377176		Human
ABCB1 (MDR-1)	mAb	FITC	Santa Cruz Biotech	sc-13131		Human
ROR1	mAb	PE	R&D Systems	FAB2000P		Human
